# A Low Frequency of Losses in 11q Chromosome Is Associated with Better Outcome and Lower Rate of Genomic Mutations in Patients with Chronic Lymphocytic Leukemia

**DOI:** 10.1371/journal.pone.0143073

**Published:** 2015-12-02

**Authors:** José Ángel Hernández, María Hernández-Sánchez, Ana Eugenia Rodríguez-Vicente, Vera Grossmann, Rosa Collado, Cecilia Heras, Anna Puiggros, Ana África Martín, Noemí Puig, Rocío Benito, Cristina Robledo, Julio Delgado, Teresa González, José Antonio Queizán, Josefina Galende, Ignacio de la Fuente, Guillermo Martín-Núñez, José María Alonso, Pau Abrisqueta, Elisa Luño, Isabel Marugán, Isabel González-Gascón, Francesc Bosch, Alexander Kohlmann, Marcos González, Blanca Espinet, Jesús María Hernández-Rivas, Grupo Cooperativo Español de Citogenética Hematológica (GCECGH) and Grupo Español de Leucemia Linfática Crónica (GELLC)

**Affiliations:** 1 Hematology Department, Hospital Universitario Infanta Leonor, Universidad Complutense de Madrid, Madrid, Spain; 2 IBSAL, IBMCC, Centro de Investigación del Cáncer, Universidad de Salamanca,CSIC, Hospital Universitario de Salamanca, Spain; 3 MLL Munich, Germany; 4 Hematology Department, Hospital General, Valencia, Spain; 5 Pathology Department, Hospital del Mar, Barcelona, Spain; 6 Hematology Department, Hospital Universitario, Salamanca, Spain; 7 Hematology Department, Hospital Clinic i Provincial, Barcelona, Spain; 8 Fundación Pública Galega de Medicina Xenómica, Santiago de Compostela, Spain; 9 Hematology Department, Hospital General, Segovia, Spain; 10 Hematology Department, Hospital del Bierzo, Ponferrada, León, Spain; 11 Hematology Department, Hospital Universitario Río Hortega, Valladolid, Spain; 12 Hematology Department, Hospital Virgen del Puerto, Plasencia, Cáceres, Spain; 13 Hematology Department, Hospital Río Carrión, Palencia, Spain; 14 Hematology Department, Hospital Vall d'Hebron, Barcelona, Spain; 15 Hematology Department, Hospital Central de Asturias, Oviedo, Spain; 16 Hematology Department, Hospital Clínico, Valencia, Spain; 17 AstraZeneca, Personalized Healthcare and Biomarkers, Innovative Medicines, Macclesfield, United Kingdom; 18 Department of Medicine, Universidad de Salamanca, Spain; University of Manitoba, CANADA

## Abstract

To analyze the impact of the 11q deleted (11q-) cells in CLL patients on the time to first therapy (TFT) and overall survival (OS), 2,493 patients with CLL were studied. 242 patients (9.7%) had 11q-. Fluorescence *in situ* hybridization (FISH) studies showed a threshold of 40% of deleted cells to be optimal for showing that clinical differences in terms of TFT and OS within 11q- CLLs. In patients with ≥40% of losses in 11q (11q-H) (74%), the median TFT was 19 months compared with 44 months in CLL patients with <40% del(11q) (11q-L) (*P*<0.0001). In the multivariate analysis, only the presence of 11q-L, mutated *IGHV* status, early Binet stage and absence of extended lymphadenopathy were associated with longer TFT. Patients with 11q-H had an OS of 90 months, while in the 11q-L group the OS was not reached (*P* = 0.008). The absence of splenomegaly (*P* = 0.02), low LDH (*P* = 0.018) or β2M (*P* = 0.006), and the presence of 11q-L (*P* = 0.003) were associated with a longer OS. In addition, to detect the presence of mutations in the *ATM*, *TP53*, *NOTCH1*, *SF3B1*, *MYD88*, *FBXW7*, *XPO1* and *BIRC3* genes, a select cohort of CLL patients with losses in 11q was sequenced by next-generation sequencing of amplicons. Eighty % of CLLs with 11q- showed mutations and fewer patients with low frequencies of 11q- had mutations among genes examined (50% *vs* 94.1%, *P* = 0.023). In summary, CLL patients with <40% of 11q- had a long TFT and OS that could be associated with the presence of fewer mutated genes.

## Introduction

In chronic lymphocytic leukemia (CLL) the presence of cytogenetic aberrations assessed by fluorescence *in situ* hybridization (FISH) influences the prognosis, in terms of time to first therapy (TFT), response to treatment, and overall survival (OS) [1]. Deletions on 11q are observed in 9–18% of CLL patients [1,2]. These patients are younger, have abdominal bulky lymph node involvement [3,4], and often show ZAP-70 expression, unmutated status of *IGHV* and shorter survival, although with a highly variable clinical outcome. The use of chemoimmunotherapy, including rituximab and cyclophosphamide to fludarabine schedules, can improve the response in patients with 11q deletion [5].

Deletions of 11q almost invariably include the ataxia telangiectasia mutated (*ATM*) gene [6]. This important tumor suppressor gene plays a crucial role in DNA repair and recombination, and regulates cell cycle progression [7]. Although mutations of this gene have been linked to poor prognosis and are associated with 11q deletions in CLL patients, due to its extreme size (62 coding exons) with lack of well characterized (hot-spot) mutations, *ATM* sequencing studies in CLL have been challenging, leaving several issues unresolved [8–10].

The application of next-generation sequencing (NGS) allows the detection of new candidate genes with frequent mutations in CLL patients as detected by whole-exome and whole-genome sequencing [11–14]. Besides *TP53* mutations [15,16], *NOTCH1* and *SF3B1*, found in around 10% of newly diagnosed CLL patients, are the most frequently mutated genes [17,18]. Patients with mutations in some of these genes have been associated with shorter TFT and OS [19,20]. Other recurrent mutations in *MYD88*, *FBXW7*, *XPO1* and *BIRC3* genes have been reported at frequencies below 10% [19,21]. Moreover, *BIRC3*, a negative regulator of NF_K_B signaling pathway, is located near to *ATM* gene, at 11q22 [9].

In the last few years, it has been reported that patients with CLL and 13q deletion may differ in their outcomes depending on the percentage of cells displaying this aberration [2,22–24]. To assess the potential prognostic value of the number of cells with deletion on 11q and to gain insight into the molecular basis of this abnormality in CLL, we have performed a multicenter study of a large series of patients diagnosed with 11q- CLL to determine whether the frequency of losses in 11q has an influence on OS and TFT. Furthermore, NGS studies were carried out, in a subset of patients, to analyze the mutational status of *ATM*, *TP53*, *NOTCH1*, *SF3B1*, *MYD88*, *FBXW7*, *XPO1* and *BIRC3* in this group of patients.

## Methods

### Patients

A total of 2,493 patients registered in the DataBase of CLL of the Spanish Group of Cytogenetics (GCECGH) and the Spanish Group of CLL (GELLC) were included. The diagnosis of CLL was made according to the International Workshop on CLL (IWCLL) criteria [25]. In all cases, an immunophenotypic analysis was performed by flow cytometry. FISH studies, including specific probes for at least the 11q22.3–23.1, 12p11.1-q11, 13q14, and 17p13 regions were carried out.

A total of 242 patients (9.7%) had an 11q deletion. The final analysis was limited to 197 cases, including 11q deletion performed at diagnosis of CLL, after excluding cases with monoclonal B-cell lymphocytosis, clonal evolution or inappropriate follow-up (Table A in S1 File). Basic clinical and biological data were recorded by reviewing the GCECGH and GELLC DataBase. The study was approved by the local ethical committees “Comité Ético de Investigación Clínica, Hospital Universitario de Salamanca”. Written informed consent was obtained from each patient before they entered the study.

### Fluorescence *in situ* hybridization (FISH)

Interphase FISH was performed in peripheral blood samples using commercially available probes for the 13q14, 12p11.1-q11 (alpha satellite), 11q22/*ATM* and 17p13/P53 regions (Vysis/Abbott Co, Abbott Park, IL, USA). Dual-color FISH using differently labeled control probes and test probes were performed. The methods used for FISH analysis have been described elsewhere [26]. Signal screening was carried out in at least 200 cells with well-delineated fluorescent spots. In cases with 11q deletion a score of ≥10% was considered positive, according to the cut-off of our laboratories.

### Next-generation sequencing analysis

A total of 25 11q- CLL patients were included in sequencing studies. Samples were obtained at diagnosis in all cases. NGS was performed using 454 Titanium Amplicon chemistry (Roche Applied Science, Penzberg, Germany) [27] to investigate the *ATM*, *TP53*, *NOTCH1*, *SF3B1*, *MYD88*, *FBXW7*, *XPO1* and *BIRC3* mutations in 11q- CLL patients. Information about primer sequences is shown in Table E in S1 File and the PCR conditions are described in Table F in S1 File. The oligonucleotide design was performed as part of the IRON-II network.

All data were generated using the GS FLX and Junior Sequencer Instrument software version 2.7 (Roche Applied Science). To detect variants, filters were set to display sequence variants occurring in more than 2% of bidirectional reads per amplicon in at least one patient. Table G in S1 File shows the median number of reads generated for each gene, allowing variants to be identified down to a detection limit of 2% [28].

Detailed methods are described in the S1 File. The sequencing data are uploaded to the Sequence Read Archive (SRA) (http://trace.ncbi.nlm.nih.gov/Traces/sra/) under accession number PRJNA297249. All the information is accessible with the following link http://www.ncbi.nlm.nih.gov/bioproject/297249.

### Statistical analysis

Statistical analysis were performed using SPSS 20.0 for Windows (SPSS, Chicago, IL, USA). TFT and OS were analyzed on the date of the initial FISH study, coinciding in all of cases with CLL diagnosis. The number of losses in 11q-deleted nuclei was divided into deciles to better define the most significant cut-off point for TFT and OS. The chi-square test was used to assess associations between categorized variables, while continuous variables were analyzed with the Mann-Whitney U test. Statistically significant variables related to TFT and OS were estimated by the Kaplan-Meier method, using the log-rank test to compare the curves of each group. Univariate and multivariate analyses of the TFT and OS employed the Cox regression method. Results were considered statistically significant for values of *P*≤0.05.

## Results

### Clinical and biological characteristics of CLL patients carrying 11q deletion

One hundred ninety-seven patients with 11q deletion were selected for the analysis. There was a predominance of males (76.6%), and the median age was 65 years (range: 28–97 years). Most patients (61%) were in Binet stage A and only 14.9% had B symptoms. In 46.4% of patients the lymphocyte blood count was ≥20 x 10^9^/L, while 31.8% and 28.9% patients, respectively, had high serum β_2_-microglobulin and high serum LDH levels. A total of 68.5% of patients had lymph node involvement, and splenomegaly was detected in 23.4% of cases. Regarding biological characteristics, *IGHV* unmutated cases were present in 66.1% of cases, while CD38 ≥30% and ZAP-70 ≥20% were detected in 55% and 55.7% of patients, respectively (Table A in S1 File).

Fifty-one patients (25.9%) had <40% of 11q-deleted cells, while 146 cases (74.1%) had ≥40% of such cells. Different cut-off points were analysed, and 40% 11q deleted nuclei was selected to better separate patients with different disease outcome. In 82 out of 197 patients (41.6%) 11q- was the sole cytogenetic aberration, while 115 patients (58.4%) had 11q deletion plus other cytogenetic abnormalities (108 cases had a 13q deletion, 14 had trisomy 12, and 6 patients showed a 17p deletion).

No significant differences in clinical or biological features were found between patients with low (<40%) and high (≥40%) frequencies of 11q- cells, except for the number of lymphocytes, Binet stage, *IGHV* mutational status, need for therapy, and death during follow-up (Table 1).

**Table 1:** Characteristics of 197 patients with 11q deletion with respect to the number of losses detected by FISH: <40% (n = 51) or ≥40% (n = 146).

Characteristic	Category	del(11q) <40%,	del(11q) ≥40%,	*P*
		N = 51 (26%)	N = 146 (74%)	* *
Age, years		62 (28–84)	65 (33–91)	**0.13**
White blood cells, range /μL		17,900 (7,800–98,100)	28,000 (6,600–365,000)	**0.008**
Lymphocytes, range /μL		12,600 (5,100–84,8500)	21,100 (5,200–364,000)	**0.007**
Lymphocytes > 20 x 10^9^/L				** **
	Yes	14	75	**0.005**
	No	35	68	
Hemoglobin, range g/dL		14 (6–17)	14 (5–17)	**0.91**
Platelet count, range /μL		195,000 (63,000–352,000)	182,000 (2,000–412,000)	**0.44**
*IGHV* (n = 56)*	* *			** **
	Mutated	9	10	**0.024**
	Unmutated	6	31	** **
ZAP-70 (n = 79)*				** **
	+	12	32	**0.44**
	-	11	24	
CD38 (n = 130)*				** **
	+	16	40	**0.139**
	-	19	56	
del(11q) as sole cytogenetic aberration				** **
	Yes	20	62	**0.74**
	No	31	84	
del(11q) + del(13q)				** **
	Yes	29	77	**0.63**
	No	22	69	
Sex				** **
	Male	40	111	**0.84**
	Female	11	35	
LDH (n = 187)*				** **
	Normal	34	99	**0.49**
	High	13	41	
β microglobulin (n = 170) *				** **
	Normal	33	80	**0.19**
	High	11	43	
Binet stage (n = 195)*				** **
	A	36	83	**0.17**
	B	10	44	** **
	C	4	14	
Lymphadenopathy (n = 193)*				** **
	No	20	41	**0.12**
	≤ 2 nodal areas	16	43	
	> 2 nodal areas	13	60	
Hepatomegaly (n = 193)*				** **
	Yes	3	15	**0.57**
	No	46	129	
Splenomegaly (n = 193)*				** **
	Yes	10	36	**0.43**
	No	39	108	
B symptoms (n = 195)*				** **
	Yes	6	23	**0.34**
	No	44	122	
Second Cancer (n = 172)*				** **
	Yes	4	22	**0.23**
	No	42	104	
Died during follow-up				** **
	Yes	10	50	**0.04**
	No	41	96	
Therapy during follow-up				** **
	Yes	27	104	**0.025**
	No	24	42	

*Number of cases.

### CLL patients with a low number of 11q- cells have a prolonged time to first therapy (TFT)

All 197 patients were evaluable for analysis of TFT, response to therapy and OS. By the time of analysis, 151/197 (76.6%) had received treatment, with a median TFT of 25 months (95% CI, 31–44 months) (Fig A in S1 File.). In terms of TFT, no differences in the group of 11q deletion as unique FISH cytogenetic aberration compared with the group of 11q deletion plus other FISH alterations were observed. A significantly longer TFT was detected in the cohort of patients with <40% of 11q deleted cells (median, 44 months; 95% CI, 33–55 months) *vs* those patients ≥40% losses in 11q (median, 19 months; 95% CI, 12–24 months) (*P*<0.0001) (. 1A). Of note, 52% of patients in the former group required treatment while 70.5% of patients with ≥40% of 11q-deleted nuclei were treated. Variables associated with a longer TFT were early clinical stage (*P* = 0.024), absence of extended lymphadenopathy (<2 node areas involved) (*P*<0.0001), absence of splenomegaly (*P* = 0.045), low serum LDH (*P* = 0.045), low serum β2M (*P* = 0.019), low CD38 expression (*P* = 0.023), low ZAP70 expression (*P* = 0.025), mutated *IGHV* status (*P*<0.0001) and del(11q) in <40% of cells (*P*<0.0001) (Table B in S1 File). In the multivariate analysis, only the presence of del(11q) in <40% of cells (Hazard Ratio, HR, 4.475; 95% CI, 1.813–7.171; *P* = 0.001), mutated *IGHV* status (HR, 3.659; *P* = 0.005), early Binet stage (HR 2.492; *P* = 0.023) and absence of extended lymphadenopathy (HR 1.854; *P* = 0.016) identified independent risk factors associated with longer TFT (Table 2).

**Table 2:** Multivariate Cox regression analysis of time to first therapy in 11q- CLL patients with respect to the number of losses detected by FISH: <40% (n = 51) or ≥40% (n = 146).*

Variable	Hazard Ratio	95% CI	*P* (log-rank test)
del(11q) <40%	4.475	1.813–7.171	**0.001**
Mutated *IGHV*	3.659	1.478–9.057	**0.005**
Early Binet stage	2.492	1.137–5.463	**0.023**
Non-extended lymphadenopathy (≤ 2 nodal areas)	1.854	1.121–3.065	**0.016**

*The following covariates were included in the final model: age, sex, Binet stage, splenomegaly, extended lymphadenopathies, LDH, β_2_ microglobulin, CD38, ZAP70, *IGHV* mutation status and percentage 11q deleted nuclei.

In addition, in patients with del(11q) as the sole cytogenetic aberration, a longer TFT was observed in patients with 11q-L (median 45 months *vs* 15 months, P < 0.0001).

### CLL patients with a low number of 11q- cells have longer overall survival (OS)

**Fig 1:**
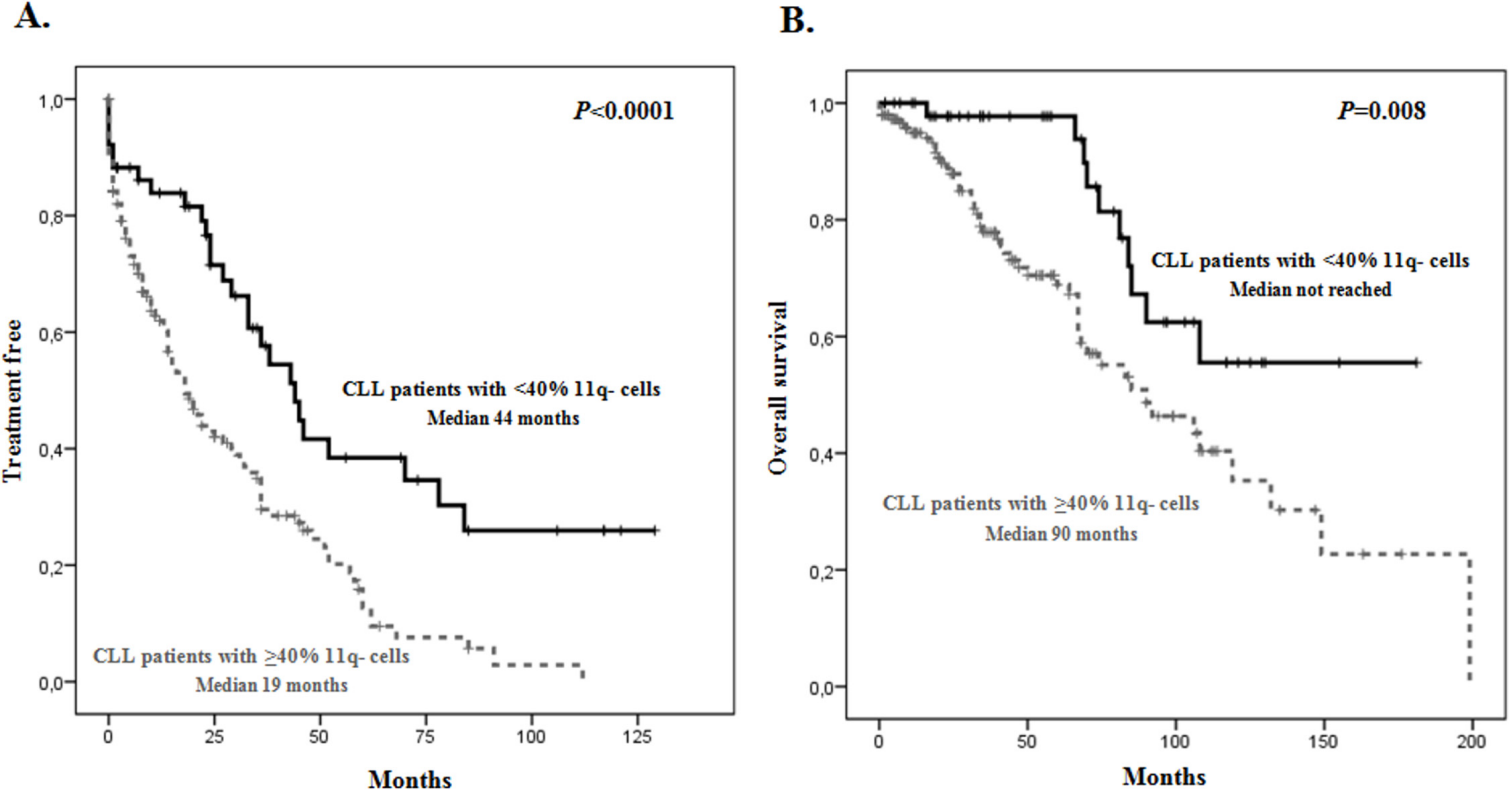
(A) Time to first therapy (TFT) and (B) overall survival (OS) of 197 patients with 11q deletion CLL and <40% or ≥40% FISH losses.

By the time of analysis, 60/197 patients (30.5%) had died. The median OS of the global series was 106 months (95% CI, 97–128 months) (Fig A in S1 File). Significantly longer survival was observed in patients with a low frequency of losses in 11q-. Thus, in patients with loss of 11q in ≥40% of cells, the OS was 90 months (95% CI, 57–123 months), while in the group with <40% of losses in 11q, the median OS had not been reached (95% CI, 114–157 months) (*P* = 0.006) (Fig 1B). In the univariate analysis, early clinical Binet stage (*P* = 0.001), asymptomatic disease (*P* = 0.034), absence of hepatomegaly (*P* = 0.025) or splenomegaly (*P*<0.0001), lymphocyte count <20 x 10^9^/L (*P* = 0.032), low serum of either LDH level (*P*<0.0001) or β_2_M (*P*<0.0001), the presence of an association of 11q deletion and 13q deletion (*P* = 0.045), and a low number (<40%) of cells with 11q- (*P* = 0.006) were associated with longer OS (Table C in S1 File). In the multivariate analysis, the variables independently related to longer OS were the absence of splenomegaly (HR, 1.786; *P* = 0.023), low serum LDH (HR, 2.076; *P* = 0.018), low serum β_2_M (HR, 2.448; *P* = 0.006) and the presence of del(11q) in <40% of cells (HR, 3.145; 95% CI, 1.474–6.691; *P* = 0.003) (Table 3).

**Table 3:** Multivariate Cox regression analysis of overall survival in 11q- CLL patients with respect to the number of losses detected by FISH: <40% (n = 51) or ≥40% (n = 146).*

Variable	Hazard Ratio	95% CI	*P* (log-rank test)
Del 11q <40%	3.145	1.474–6.691	**0.003**
Low serum β_2_ microglobulin	2.448	1.260–4.753	**0.006**
Low serum LDH	2.076	1.061–4.064	**0.018**
Absence of splenomegaly	1.786	1.044–4.481	**0.023**

*The following covariates were included in the final model: age, sex, Binet stage, splenomegaly, extended lymphadenopathies, LDH, β_2_ microglobulin, CD38, ZAP70, *IGHV* mutation status and percentage 11q deleted nuclei.

Regarding the patients with del(11q) as the unique cytogenetic aberration, a longer OS was observed in patients with 11q-L (median not reached *vs* 70 months, P = 0.007)

The analyses of other cut-offs for the number of 11q- cells (<40% *vs* 40–59% *vs* ≥60%) showed similar results for TFT and OS (Fig B in S1 File).

### Biallelic inactivation of the *ATM* gene is observed in one-third of 11q- CLL patients

**Fig 2:**
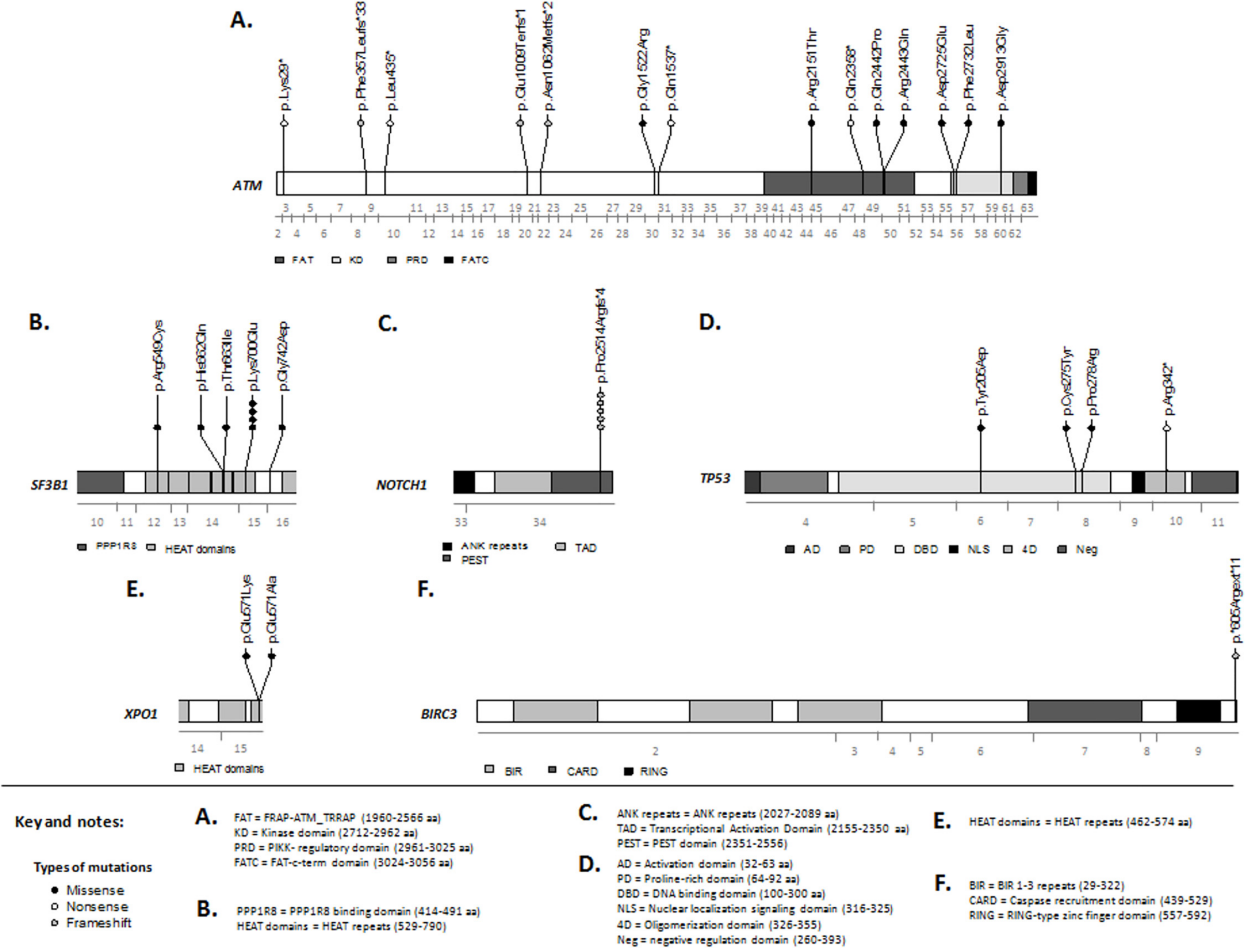
Localization and frequencies of mutations in *ATM*, *SF3B1*, *NOTCH1*, *TP53*, *XPO1* and *BIRC3* genes. Mutations are indicated at the amino-acid level; each detected alteration is represented by a dot. Mutation type is represented by a dark (missense), white (nonsense) or grey (frameshift) dot. The sequenced exons for each gene are represented with a grey line below each schematic protein organization.

*ATM* mutations were found in eight (32%) of 25 patients with 11q-. In total, 14 different mutations were detected by *ATM* molecular mutation screening: 11 point mutations (7 missense and 4 nonsense; 78.6%) and three frameshift mutations (2 deletions and 1 insertion; 21.4%). These mutations are shown in Fig 2 and listed in Table 4. All patients with mutated *ATM* had at least one truncating or damaging mutation. Interestingly, four of the eight patients with *ATM* mutations carried more than one type of mutation. Thus, two patients with *ATM* mutations carried two different mutations while two other patients with *ATM* mutations carried three mutations. It is of note that the patients with several *ATM* mutations had different mutational loads, suggesting the presence of independent clones or clonal evolution with the acquisition of a second mutation. However, it could not be confirmed whether the mutations from one patient belonged to different clones, since they were located on distinct sequencing reads in different amplicons.

**Table 4:** *ATM* mutations in 25 CLL patients with del(11q).

Patient ID	FISH			*ATM* mutations							
	% 11q-	11q- Group	Other abnormalities	Mutational load (%)	Sequence change	Exon	Protein change	Domain protein	Type of mutation	Consequence (SIFT)	Reported previously
14	27	11q-L	30% 13q-	13	c.85A>T	3	p.Lys29X	-	nonsense	T	-
14	27	11q-L	30% 13q-	24	c.6452G>C	44	p.Arg2151Thr	FAT	missense	NT (Tolerated)	-
2	33	11q-L	-	56.5	c.7325A>C	50	p.Gln2442Pro	FAT	missense	NT (Damaging)	1, 2, 3, 4
3	38	11q-L	89% 13q-	5.5	c.3024insT	20	p.Gln1009fs	-	frameshift	T	-
3	38	11q-L	89% 13q-	14.5	c.7072C>T	48	p.Gln2358X	FAT	nonsense	T	-
18	70	11q-H	37.5% 13q-	7	c.1304T>A	10	p.Leu435X	-	nonsense	T	-
18	70	11q-H	37.5% 13q-	2	c.4564G>C	30	p.Gly1522Arg	-	missense	NT (Damaging)	-
18	70	11q-H	37.5% 13q-	26	c.7328G>A	50	p.Arg2443Gln	FAT	missense	NT (Damaging)	5, 6
6	73	11q-H	-	43	c.3185delA	22	p.Asn1062fs	-	frameshift	T	-
6	73	11q-H	-	7	c.8196C>A	56	p.Phe2732Leu	PI3K	missense	NT (Damaging)	-
6	73	11q-H	-	3.5	c.8738A>G	60	p.Asp2913Gly	PI3K	missense	NT (Damaging)	-
23	82.5	11q-H	-	14	c.1067delT	9	p.Phe357LeufsX33	-	frameshift	T	-
16	83	11q-H	56% 13q-	92.5	c.8175T>A	56	p.Asp2725Glu	PI3K	missense	NT (Damaging)	-
15	88.5	11q-H	85% 13q-	79.5	c.4609C>T	30	p.Gln1537X	-	nonsense	T	-

11q-L: 11q- with <40% 11q-

11q-H: ≥40% 11q-

T: Truncating mutation

NT: Non-truncating mutation

1 Fujimoto A et al Nature Genetics 2012

2 Ding et al Nature 2008

3 Davies H et al Cancer Research 2005

4 Vorechovsky Nature Genetics 1997

5 Yip S et al. J Pathol 2012

6 Greiner TC et al Proc Natl Acad Sci USA 2006.

All the mutations were located in exons 3–60, between amino acids 29 and 2913, and involved the FAT and PI3K domains of the ATM protein. Five mutations resulted in a truncated form of the ATM protein (termed ‘truncating’) while the remaining mutations resulted in the expression of the full-length, but mutated form of the ATM protein (termed ‘non-truncating’). These missense mutations were analyzed with SIFT algorithms, which predicted six of them to be damaging. The median mutational burden was 14.3% (range, 2.0–92.5%). Eight of 14 (57.1%) variants had a mutational load of ≤15% and thus would not have been detected by capillary Sanger sequencing.

The TFT and OS were assessed in all patients and related to the mutational status of the *ATM* gene. There was no difference in TFT or OS between 11q- patients with and without *ATM* mutations (*P*<0.970 and *P*<0.623, respectively). Interestingly, bivariate analyses indicated that the presence of *ATM* mutations was a useful characteristic for identifying CLL patients with a different TFT in the subgroup of CLL patients with low frequencies of 11q-. Thus, CLL with *ATM* mutations had a shorter TFT than patients without *ATM* mutations (36 *vs* 46 months; *P* = 0.046). By contrast, 11q patients harboring ≥40% of 11q- had shorter TFT independently of *ATM* mutational status. Moreover, there was no significant association between the presence of an *ATM* mutation and other clinical or biological prognostic factors (Table D in S1 File).

### *SF3B1* is a frequently mutated gene in CLL patients with 11q-

Mutations in *TP53*, *NOTCH1*, *SF3B1*, *MYD88*, *FBXW7*, *XPO1* and *BIRC3* were analyzed in the entire cohort of 25 CLL patients. In total, 20 mutations were detected: eight patients had *SF3B1* mutations, five cases had mutations in *NOTCH1*, four in *TP53*, two in *XPO1*, while one CLL had a mutation in *BIRC3*. Most of them had previously been described as mutations in the COSMIC database. The median mutational burden was 27% (range, 3–81%). In 7/20 (35%) variants the mutation load was ≤15%. The frequency of 11q- CLL with associated mutations was 8/25 (32%) for *SF3B1*, 5/25 (20%) for *NOTCH1*, 4/25 (16%) for *TP53*, 2/25 (8%) for *XPO1* and 1/25 (4%) for *BIRC3*. These mutations are shown in Fig 2 and listed in Table 5. Confirming previously published sequencing data, the most frequent *SF3B1* mutation was p.Lys700Glu (4/8, 50%) while the presence of p.Pro2514Argfs*4 was the most frequent *NOTCH1* mutation (5/5, 100%). In addition, 50% of the 11q- CLL patients with *TP53* mutations also showed 17p-.

**Table 5:** Mutations in other genes in 25 CLL patients with del(11q).

Patient ID	FISH			Mutations					
	% 11q-	11q- group	Other abnormalities	Gene	Mutational load (%)	Sequence change	Exon	Protein change	COSMIC database
10	26	11q-L	-	*SF3B1*	17	c.2225G>A	16	p.Gly742Asp	COSM145923
14	27	11q-L	30% 13q-	*NOTCH1*	6	c.7541_7542delCT	34	p.Pro2514ArgfsX4	COSM12774
22	48	11q-H	-	*SF3B1*	4	c.2098A>G	15	p.Lys700Glu	COSM84677
22	48	11q-H	-	*TP53*	28	c.824G>A	8	p.Cys275Tyr	COSM10893
21	48.5	11q-H	81.5% 13q-	*SF3B1*	25	c.1645C>T	12	p.Arg549Cys	COSM1014502
21	48.5	11q-H	81.5% 13q-	*TP53*	16	c.1024C>T	10	p.Arg342X	COSM11073
25	62	11q-H	-	*NOTCH1*	68.5	c.7541_7542delCT	34	p.Pro2514ArgfsX4	COSM12774
18	70	11q-H	37.5% 13q-	*SF3B1*	34	c.2098A>G	15	p.Lys700Glu	COSM84677
6	73	11q-H	-	*SF3B1*	39	c.2098A>G	15	p.Lys700Glu	COSM84677
6	73	11q-H	-	*NOTCH1*	4	c.7541_7542delCT	34	p.Pro2514ArgfsX4	COSM12774
20	78	11q-H	63% 13q-	*SF3B1*	46.5	c.1988C>T	14	p.Thr663Ile	COSM145921
16	83	11q-H	56% 13q-	*SF3B1*	4	c.2098A>G	15	p.Lys700Glu	COSM84677
13	83	11q-H	86% 13q-	*TP53*	7.5	c.613T>G	6	p.Tyr205Asp	COSM43844
9	83.5	11q-H	61% 13q-	*NOTCH1*	3	c.7541_7542delCT	34	p.Pro2514ArgfsX4	COSM12774
5	84.5	11q-H	87% 17p-	*NOTCH1*	45	c.7541_7542delCT	34	p.Pro2514ArgfsX4	COSM12774
19	84.5	11q-H	85% 13q-	*XPO1*	47.5	c.1711G>A	15	p.Glu571Lys	COSM96797
4	89	11q-H	87% 17p-	*TP53*	81	c.833C>G	8	p.Pro278Arg	COSM10887
4	89	11q-H	87% 17p-	*BIRC3*	12	c.1813T>C	9	p.X605ArgextX11	-
1	90	11q-H	87%13q-	*SF3B1*	50.5	c.1986C>G	14	p.His662Gln	COSM110692
17	97	11q-H	97% 13q-	*XPO1*	51	c.1712A>C	15	p.Glu571Ala	COSM1291526

In terms of prognostic relevance, significant differences were observed only in TFT between 11q- patients with and without *NOTCH1* mutations (5 *vs* 36 months; *P* = 0.031) and in OS between patients with and without *TP53* mutations (1 *vs* 197 months; *P*<0.003) (Fig C in S1 File).

### Genetic mutations are associated with a higher percentage of 11q- cells

**Fig 3:**
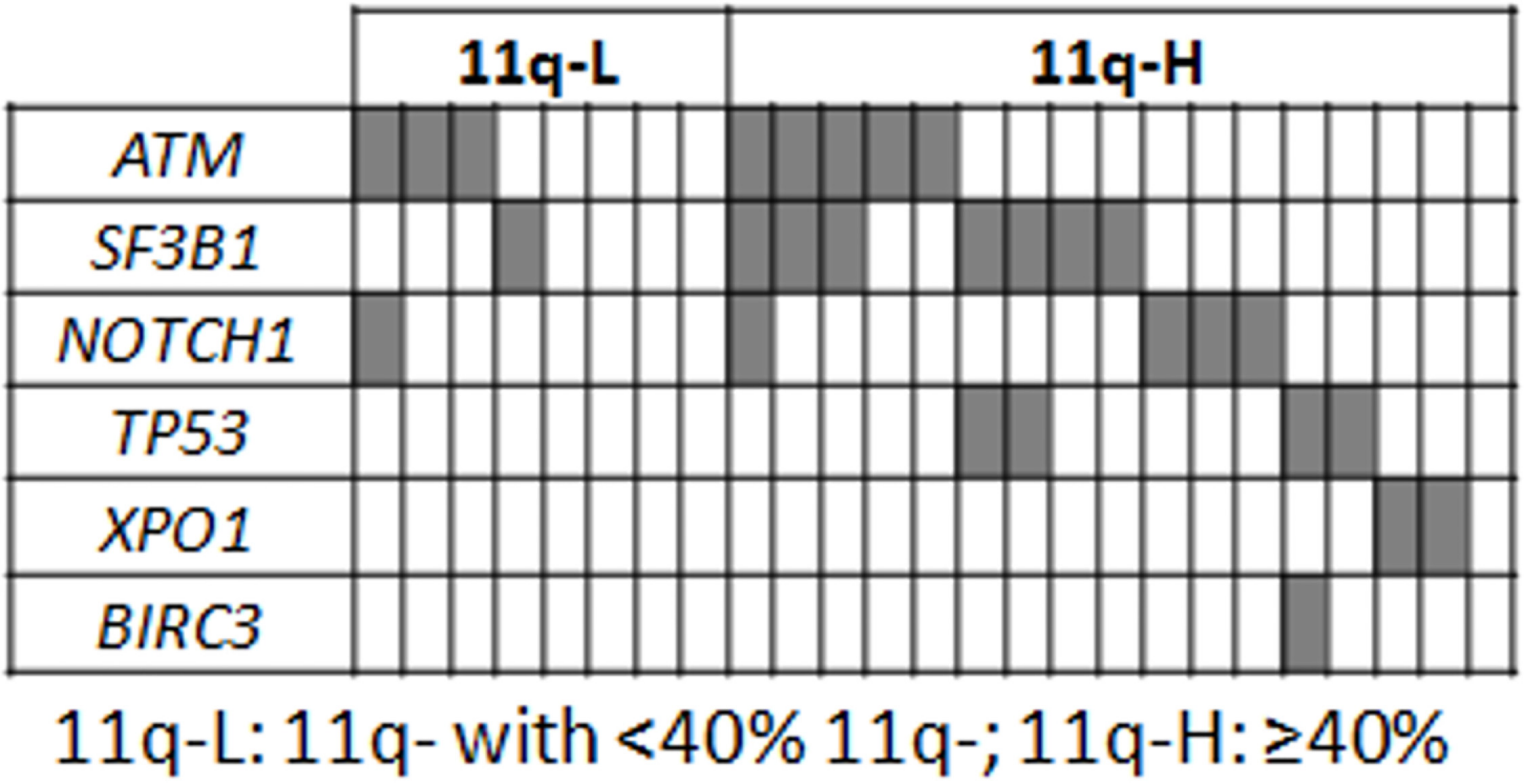
Distribution of mutations of *ATM*, *SF3B1*, *NOTCH1*, *TP53*, *XPO1* and *BIRC3* among 11q- CLL patients with respect to the percentage of 11q- cells. In the heat maps, rows correspond to identical genes, and columns represent individual patients color-coded on the basis of gene status (white: wild type; grey: mutated gene).

As a next step towards understanding the clinical differences within the 11q- subgroup, the association between the presence of genetic mutations and the percentage of 11q- cells in CLL patients was examined. The incidence of *ATM* mutations was similar in the two groups. Thus, 29.4% of patients with a high frequency of 11q- exhibited *ATM* mutations while 37.5% of patients with a low frequency of 11q losses had *ATM* mutations (*P* = 0.513). However, considering the mutations of all the genes analyzed, fewer patients with low frequencies of 11q- had mutations among genes examined compared with the subgroup of a high number of losses 11q- (4/8, 50% *vs* 16/17, 94.1%; *P* = 0.023) (Fig 3). Interestingly, among the CLL patients without a gene mutation, the median proportion of the CLL tumor population with an 11q deletion was significantly lower than that of CLL patients with mutated genes (20.5%, range, 12–71.5% *vs* 80.3%, range, 26–97%; *P* = 0.007). *TP53* mutations were present only in patients with a high frequency of 11q- cells.

## Discussion

In this study, we analyzed the outcome of 11q-deleted CLL patients at diagnosis with respect to TFT and OS, and the presence of mutations in the most relevant genes to date in CLL. The importance of the percentage of cells displaying a genetic aberration determined by FISH, as an independent prognostic factor in CLL, has recently been recognized in 17p-, 13q- and +12 [2,22–24,29,30], whereby a high number of cells with 13q deletion has been associated with a worse outcome [2,22–24]. In fact, patients displaying a high degree of infiltration of 13q- had an intermediate prognosis, with a shorter time to first therapy and survival than those with normal cytogenetics or trisomy 12 [2,22–24]. In addition, patients with fewer losses in 17p or patients with a low number of trisomy 12 have a better outcome [29,31]. Several reports have shown the 11q deletion to be a factor predicting poor prognosis in CLL [1,25,32]. In the present study, we have confirmed these previous observations, whereby CLL patients with 11q- had a median time of 2 years to receipt of first therapy and an overall survival of 8 years. Although the present study is a retrospective and multicenter analysis of a series of CLLs, the characteristics of the patients agree real-world basis. Thus, a median age of 65 years, predominance of male sex (76%) and frequent lymphadenopathy (69%) were observed, as previously described [1]. In addition, 66% of patients had unmutated *IGHV* status with more than 50% of cases expressing CD38 and/or ZAP-70. Of note, we have observed that the number of cells carrying 11q- influences the disease outcome in terms of time to first therapy and overall survival. These results are consistent with those of two recently published series that reported a longer TFT in patients with 11q deletion and a low number of losses [33,34]. However, one of these studies found no improvement in the overall survival in this group of patients [34]. Therefore, the present study confirms that patients with 11q deletion do not comprise a homogeneous subgroup. We identified a threshold of 40% of deleted cells to be optimal for showing that a low number of losses in 11q is associated with a better outcome in terms of TFT and OS. However, the use of other cut-off points (i.e., <40% *vs* 40–59% *vs* ≥60%) yielded similar results.

Interestingly, this study showed that CLL with losses in 11q with early clinical stage, mutated *IGHV* status and/or a low number of losses in the 11q chromosome had a TFT of more than two years compared with cases with >40% of cells with 11q deletion (median, 44 *vs* 19 months), similar to previously reported results [34]. Therefore, we identified the presence of advanced clinical stages, unmutated *IGHV*, and a high number of losses in 11q as the main predictors indicating therapy in the group of CLL displaying 11q-.

Moreover, CLL patients with a lower percentage of 11q deletion had a better OS than those with ≥40% of 11q-deleted nuclei (median, not reached *vs* 90 months), with an estimated 3 years longer survival in patients with less than 40% of 11q deletions. In addition, clinical (absence of splenomegaly) or biological variables (low 11q-, low LDH and β_2_M) were included in the final multivariate model of OS. Therefore, the assessment of the number of 11q- cells should be included to better define survival in patients with CLL displaying this genetic abnormality and could be incorporated into the design of clinical trials to define their influence on the response to new therapies and on survival.

To better define the molecular characteristics of the CLL patients displaying losses in 11q, a mutational study performed by next-generation sequencing, including the most frequently mutated genes in CLL, was carried out. The results showed that patients with a low number of 11q losses displayed fewer mutations. Interestingly, *ATM* mutations were present in both cohorts of 11q- patients at a similar frequency to those previously reported [9,35–37]. We found no significant impact of *ATM* mutations on prognosis for all the 11q- patients, as described by other authors [9,37]. However, focusing on the group of patients with a low number of 11q losses, *ATM* mutations were useful for identifying CLL patients with a shorter TFT. Thus, our results suggest that the integration of molecular markers, such as *ATM* mutations, and the FISH analysis, in patients showing loss of 11q could provide a better prognostic stratification than has been recently demonstrated in other CLL patients [20].

The presence of gene mutations has been widely demonstrated in CLL [11–14]. Mutations of *TP53*, *NOTCH1*, *SF3B1* and *BIRC3* are known to be associated with a worse prognosis, while mutations in *MYD88* are related to a better outcome [18,19,21,38,39]. However, some of the results concerning the incidence and independent prognostic value of these mutations are controversial [12,14]. We observed a higher percentage of CLL patients with *XPO1* mutations in our cohort of patients than in those described by others [12,13,19,40]. However, it should be taken in account the limitation of the size of our sequencing samples cohort. Consistent with previous studies, mutations of *NOTCH1* and *TP53* occurred as mutually exclusive events [13,15]. Of note, *TP53* mutations were only detected in patients with a high frequency of 11q losses. Furthermore, *NOTCH1* and *SF3B1* mutations were more frequent in this group of 11q- CLLs (Fig 3). Therefore, our study provides new insights into the molecular basis of the worse outcome of CLL patients who have losses in 11q. It should be noted that the high frequency of gene mutations did not involve the *ATM* gene, suggesting that the clonal evolution (heterogeneity) affecting any CLL-related gene could be the basis of the dismal prognosis of patients with a high frequency of 11q- [12,40,41].

In summary, our results suggest that in patients with CLL, the frequency of 11q-deleted cells influences the clinical outcome, and a low number of 11q- is associated with a longer time to progression and overall survival. In addition, this study shows that fewer CLL patients with low frequencies of 11q- had mutations among genes examined. Our findings, derived from a large retrospective cohort of CLL patients from several Spanish institutions, need to be validated in prospective clinical trials.

## Supporting Information

S1 File Supplementary methods data. Characteristics of the series of 197 CLL patients with 11q deletion (**Table A**). Univariate analysis of time to first therapy in 11q- CLL patients with respect to the number of losses detected by FISH: <40% (n = 51) or ≥40% (n = 146) (**Table B**). Univariate analysis of overall survival in 11q- CLL patients with respect to the number of losses detected by FISH: <40% (n = 51) or ≥40% (n = 146) (**Table C**). Main clinical and biological characteristics of 25 CLL patients with 11q- with respect to *ATM* mutational status (**Table D**). PCR primers used for next-generation sequencing studies (**Table E**). A: PCR amplification protocol for *ATM*. B. B: PCR amplification protocol for the remaining genes (**Table F**). Median frequency of reads generated by next-generation sequencing (NGS) (**Table G**). A. Time to first therapy (TFT) and B. Overall survival (OS) of the global series of 197 CLL patients with 11q deletion (**Fig A**), A. Time to first therapy (TFT) and B. Overall survival (OS) of patients with CLL and 11q deletion and a percentage of FISH losses <40%, 41–59% or ≥60% (**Fig B**). Kaplan-Meier plots of time to first therapy (TFT) (A) and overall survival (OS) (B) from diagnosis for 11q- CLL patients sequenced for *NOTCH1* and *TP53*, respectively (**Fig C**)
(DOC)

## References

[1] Dohner H, Stilgenbauer S, Benner A, Leupolt E, Krober A, Bullinger L, et al Genomic aberrations and survival in chronic lymphocytic leukemia. N Engl J Med. 2000;343: 1910–6. 11136261 10.1056/NEJM200012283432602

[2] Hernandez JA, Rodriguez AE, Gonzalez M, Benito R, Fontanillo C, Sandoval V, et al A high number of losses in 13q14 chromosome band is associated with a worse outcome and biological differences in patients with B-cell chronic lymphoid leukemia. Haematologica. 2009;94: 364–71. 10.3324/haematol.13862 19252174 PMC2649343

[3] Dohner H, Stilgenbauer S, James MR, Benner A, Weilguni T, Bentz M, et al 11q deletions identify a new subset of B-cell chronic lymphocytic leukemia characterized by extensive nodal involvement and inferior prognosis. Blood. 1997;89: 2516–22. 9116297

[4] Greipp PT, Smoley SA, Viswanatha DS, Frederick LS, Rabe KG, Sharma RG, et al Patients with chronic lymphocytic leukaemia and clonal deletion of both 17p13.1 and 11q22.3 have a very poor prognosis. Br J Haematol. 2013;163: 326–33. 10.1111/bjh.12534 24032430 PMC3907074

[5] Tsimberidou AM, Tam C, Abruzzo LV, O'Brien S, Wierda WG, Lerner S, et al Chemoimmunotherapy may overcome the adverse prognostic significance of 11q deletion in previously untreated patients with chronic lymphocytic leukemia. Cancer. 2009;115: 373–80. 10.1002/cncr.23993 19117034 PMC4404627

[6] Stilgenbauer S, Liebisch P, James MR, Schroder M, Schlegelberger B, Fischer K, et al Molecular cytogenetic delineation of a novel critical genomic region in chromosome bands 11q22.3–923.1 in lymphoproliferative disorders. P Proc Natl Acad Sci U S A. 1996;93: 11837–41. 10.1073/pnas.93.21.11837 PMC38145 8876224

[7] Negrini S, Gorgoulis VG, Halazonetis TD. Genomic instability—an evolving hallmark of cancer. Nat Rev Mol Cell Biol. 2010;11: 220–8. 10.1038/nrm2858 20177397

[8] Bullrich F, Rasio D, Kitada S, Starostik P, Kipps T, Keating M, et al ATM mutations in B-cell chronic lymphocytic leukemia. Cancer research. 1999;59: 24–7. 9892178

[9] Rose-Zerilli MJ, Forster J, Parker H, Parker A, Rodriguez AE, Chaplin T, et al ATM mutation rather than BIRC3 deletion and/or mutation predicts reduced survival in 11q-deleted chronic lymphocytic leukemia: data from the UK LRF CLL4 trial. Haematologica. 2014;99:736–42. 10.3324/haematol.2013.098574 24584352 PMC3971084

[10] Schaffner C, Stilgenbauer S, Rappold GA, Dohner H, Lichter P. Somatic ATM mutations indicate a pathogenic role of ATM in B-cell chronic lymphocytic leukemia. Blood. 1999;94: 748–53. 10397742

[11] Fabbri G, Rasi S, Rossi D, Trifonov V, Khiabanian H, Ma J, et al Analysis of the chronic lymphocytic leukemia coding genome: role of NOTCH1 mutational activation. J Exp Med. 2011;208: 1389–401. 10.1084/jem.20110921 21670202 PMC3135373

[12] Puente XS, Pinyol M, Quesada V, Conde L, Ordonez GR, Villamor N, et al Whole-genome sequencing identifies recurrent mutations in chronic lymphocytic leukaemia. Nature. 2011;475(7354):101–5. 10.1038/nature10113 21642962 PMC3322590

[13] Quesada V, Conde L, Villamor N, Ordonez GR, Jares P, Bassaganyas L, et al Exome sequencing identifies recurrent mutations of the splicing factor SF3B1 gene in chronic lymphocytic leukemia. Nat genet. 2012;44: 47–52. 10.1038/ng.1032 22158541

[14] Wang L, Lawrence MS, Wan Y, Stojanov P, Sougnez C, Stevenson K, et al SF3B1 and other novel cancer genes in chronic lymphocytic leukemia. N Engl J Med. 2011;365: 2497–506. 10.1056/NEJMoa1109016 22150006 PMC3685413

[15] Dicker F, Herholz H, Schnittger S, Nakao A, Patten N, Wu L, et al The detection of TP53 mutations in chronic lymphocytic leukemia independently predicts rapid disease progression and is highly correlated with a complex aberrant karyotype. Leukemia. 2009;23: 117–24. 10.1038/leu.2008.274 18843282

[16] Rossi D, Cerri M, Deambrogi C, Sozzi E, Cresta S, Rasi S, et al The prognostic value of TP53 mutations in chronic lymphocytic leukemia is independent of Del17p13: implications for overall survival and chemorefractoriness. Clin Cancer Res. 2009;15: 995–1004. 10.1158/1078-0432.CCR-08-1630 19188171

[17] Mansouri L, Cahill N, Gunnarsson R, Smedby KE, Tjonnfjord E, Hjalgrim H, et al NOTCH1 and SF3B1 mutations can be added to the hierarchical prognostic classification in chronic lymphocytic leukemia. Leukemia. 2013;27: 512–4. 10.1038/leu.2012.307 23138133

[18] Rossi D, Rasi S, Fabbri G, Spina V, Fangazio M, Forconi F, et al Mutations of NOTCH1 are an independent predictor of survival in chronic lymphocytic leukemia. Blood. 2012;119: 521–9. 10.1182/blood-2011-09-379966 22077063 PMC3257017

[19] Jeromin S, Weissmann S, Haferlach C, Dicker F, Bayer K, Grossmann V, et al SF3B1 mutations correlated to cytogenetics and mutations in NOTCH1, FBXW7, MYD88, XPO1 and TP53 in 1160 untreated CLL patients. Leukemia. 2014;28: 108–17. 10.1038/leu.2013.263 24113472

[20] Rossi D, Rasi S, Spina V, Bruscaggin A, Monti S, Ciardullo C, et al Integrated mutational and cytogenetic analysis identifies new prognostic subgroups in chronic lymphocytic leukemia. Blood. 2013;121: 1403–12. 10.1182/blood-2012-09-458265 23243274 PMC3578955

[21] Rossi D, Fangazio M, Rasi S, Vaisitti T, Monti S, Cresta S, et al Disruption of BIRC3 associates with fludarabine chemorefractoriness in TP53 wild-type chronic lymphocytic leukemia. Blood. 2012;119: 2854–62. 10.1182/blood-2011-12-395673 22308293

[22] Dal Bo M, Rossi FM, Rossi D, Deambrogi C, Bertoni F, Del Giudice I, et al 13q14 deletion size and number of deleted cells both influence prognosis in chronic lymphocytic leukemia. Genes, chromosomes cancer. 2011;50: 633–43. 10.1002/gcc.20885 21563234

[23] Puiggros A, Delgado J, Rodriguez-Vicente A, Collado R, Aventin A, Luno E, et al Biallelic losses of 13q do not confer a poorer outcome in chronic lymphocytic leukaemia: analysis of 627 patients with isolated 13q deletion. Br J Haematol. 2013;163: 47–54. 10.1111/bjh.12479 23869550

[24] Van Dyke DL, Shanafelt TD, Call TG, Zent CS, Smoley SA, Rabe KG, et al A comprehensive evaluation of the prognostic significance of 13q deletions in patients with B-chronic lymphocytic leukaemia. Br J Haematol. 2010;148: 544–50. 10.1111/j.1365-2141.2009.07982.x 19895615 PMC2866061

[25] Hallek M, Cheson BD, Catovsky D, Caligaris-Cappio F, Dighiero G, Dohner H, et al Guidelines for the diagnosis and treatment of chronic lymphocytic leukemia: a report from the International Workshop on Chronic Lymphocytic Leukemia updating the National Cancer Institute-Working Group 1996 guidelines. Blood. 2008;111: 5446–56. 10.1182/blood-2007-06-093906 18216293 PMC2972576

[26] Gonzalez MB, Hernandez JM, Garcia JL, Lumbreras E, Castellanos M, Hernandez JM, et al The value of fluorescence in situ hybridization for the detection of 11q in multiple myeloma. Haematologica. 2004;89: 1213–8. 15477206

[27] Margulies M, Egholm M, Altman WE, Attiya S, Bader JS, Bemben LA, et al Genome sequencing in microfabricated high-density picolitre reactors. Nature. 2005;437: 376–80. 16056220 10.1038/nature03959 PMC1464427

[28] Grossmann V, Roller A, Klein HU, Weissmann S, Kern W, Haferlach C, et al Robustness of amplicon deep sequencing underlines its utility in clinical applications. J Mol Diagn. 2013;15: 473–84. 10.1016/j.jmoldx.2013.03.003 23680131

[29] Gonzalez-Gascon YMI, Hernandez-Sanchez M, Rodriguez-Vicente AE, Sanzo C, Aventin A, Puiggros A, et al A high proportion of cells carrying trisomy 12 is associated with a worse outcome in patients with chronic lymphocytic leukemia. Hematol Oncol. 2015 10.1002/hon.2196 [Epub ahead of print] 25689772

[30] Rodriguez-Vicente AE, Diaz MG, Hernandez-Rivas JM. Chronic lymphocytic leukemia: a clinical and molecular heterogenous disease. Cancer genet. 2013;206: 49–62. 10.1016/j.cancergen.2013.01.003 23531595

[31] Tam CS, Shanafelt TD, Wierda WG, Abruzzo LV, Van Dyke DL, O'Brien S, et al De novo deletion 17p13.1 chronic lymphocytic leukemia shows significant clinical heterogeneity: the M. D. Anderson and Mayo Clinic experience. Blood. 2009;114: 957–64. 10.1182/blood-2009-03-210591 19414856 PMC4916942

[32] Catovsky D, Richards S, Matutes E, Oscier D, Dyer MJ, Bezares RF, et al Assessment of fludarabine plus cyclophosphamide for patients with chronic lymphocytic leukaemia (the LRF CLL4 Trial): a randomised controlled trial. Lancet. 2007;370: 230–9. 17658394 10.1016/S0140-6736(07)61125-8

[33] Jain P, Keating M, Thompson P, Trinh L, Wang X, Wierda W, et al High FISH percentage of deletion 11q in patients with chronic lymphocytic leukemia is an independent predictor of adverse outcome. Am J Hematol. 2015;90: 471–7 10.1002/ajh.23978 25683856 PMC4521389

[34] Marasca R, Maffei R, Martinelli S, Fiorcari S, Bulgarelli J, Debbia G, et al Clinical heterogeneity of de novo 11q deletion chronic lymphocytic leukaemia: prognostic relevance of extent of 11q deleted nuclei inside leukemic clone. Hematol Oncol. 2013;31: 88–95. 10.1002/hon.2028 23027683

[35] Austen B, Skowronska A, Baker C, Powell JE, Gardiner A, Oscier D, et al Mutation status of the residual ATM allele is an important determinant of the cellular response to chemotherapy and survival in patients with chronic lymphocytic leukemia containing an 11q deletion. J Clin Oncol. 2007;25: 5448–57. 17968022 10.1200/JCO.2007.11.2649

[36] Navrkalova V, Sebejova L, Zemanova J, Kminkova J, Kubesova B, Malcikova J, et al ATM mutations uniformly lead to ATM dysfunction in chronic lymphocytic leukemia: application of functional test using doxorubicin. Haematologica. 2013;98: 1124–31. 10.3324/haematol.2012.081620 23585524 PMC3696617

[37] Skowronska A, Parker A, Ahmed G, Oldreive C, Davis Z, Richards S, et al Biallelic ATM inactivation significantly reduces survival in patients treated on the United Kingdom Leukemia Research Fund Chronic Lymphocytic Leukemia 4 trial. J Clin Oncol. 2012;30: 4524–32. 10.1200/JCO.2011.41.0852 23091097

[38] Oscier DG, Rose-Zerilli MJ, Winkelmann N, Gonzalez de Castro D, Gomez B, Forster J, et al The clinical significance of NOTCH1 and SF3B1 mutations in the UK LRF CLL4 trial. Blood. 2013;121: 468–75. 10.1182/blood-2012-05-429282 23086750

[39] Weissmann S, Roller A, Jeromin S, Hernandez M, Abaigar M, Hernandez-Rivas JM, et al Prognostic impact and landscape of NOTCH1 mutations in chronic lymphocytic leukemia (CLL): a study on 852 patients. Leukemia. 2013;27: 2393–6. 10.1038/leu.2013.218 23860447

[40] Landau DA, Carter SL, Stojanov P, McKenna A, Stevenson K, Lawrence MS, et al Evolution and impact of subclonal mutations in chronic lymphocytic leukemia. Cell. 2013;152: 714–26. 10.1016/j.cell.2013.01.019 23415222 PMC3575604

[41] Vogelstein B, Papadopoulos N, Velculescu VE, Zhou S, Diaz LA Jr., Kinzler KW. Cancer genome landscapes. Science. 2013;339: 1546–58. 10.1126/science.1235122 23539594 PMC3749880

