# Supplementary material for: A Low Frequency of Losses in 11q Chromosome Is Associated with Better Outcome and Lower Rate of Genomic Mutations in Patients with Chronic Lymphocytic Leukemia
Source: PLoS One. 2015 Dec 2;10(11):e0143073. doi: 10.1371/journal.pone.0143073 (PMC4667902; doi:10.1371/journal.pone.0143073)

**Supplementary Material**

**Supplementary Methods**

**Next-generation sequencing (NGS) analysis**

Genomic DNA was extracted from CLL fixed cells from peripheral blood of 25 11q- CLL patients using a QIAamp DNA Mini Kit (Qiagen, Valencia, CA, USA), following the manufacturer’s protocol. Due to the large quantity of DNA required for this study, 100-ng inputs of samples were amplified with the REPLI-g Midi Kit (Qiagen, Valencia, CA, USA).^1^ To ensure good quality, amplified DNA was quantified using a standardized PicoGreen fluorescence assay (LifeTechnologies, Carlsbad, CA, USA). The integrity of the DNA was visually inspected on a 1% agarose gel.

NGS was performed using 454 Titanium Amplicon chemistry (Roche Applied Science, Penzberg, Germany)^2^ to investigate the *ATM*, *TP53, NOTCH1*, *SF3B1, MYD88, FBXW7, XPO1* and *BIRC3* mutations. The eight candidate genes were screened for mutations as follows: 62 *ATM* coding exons (Transcript-ID ENST00000278616) represented by 65 amplicons; exons 4-10 of *TP53* (8 amplicons, Transcript-ID ENST00000269305); the C-terminal PEST domain (exons 33-34) of *NOTCH1* (7 amplicons, Transcript-ID ENST00000277541); in exons 10-16 of *SF3B1* (7 amplicons, Transcript-ID ENST00000335508); exons 4 and 5 of *MYD88* (2 amplicons, Transcript-ID ENST00000396334); exons 8-12 of *FBXW7* (5 amplicons, Transcript-ID ENST00000281708); exons 14 and 15 of *XPO1* (2 amplicons, Transcript-ID ENST00000401558); and exons 2-9 of *BIRC3* (11 amplicons, Transcript-ID ENST00000263464). Information about primer sequences is shown in S5 Table. The oligonucleotide design was performed as part of the IRON-II network.

A total of 96 amplicon preparations across 25 samples (2400 individual PCR reactions) were processed using the FastStart High Fidelity PCR System kit (Roche Applied Science). The PCR conditions are described in S6 Table. After all 25 samples had been prepared in 96-well plates, each PCR product was individually purified using Agencourt AMPure XP beads (Beckman Coulter, Krefeld, Germany) and checked by electrophoresis on 1.2% agarose gels.

A single patient-specific *ATM* library was generated for each patient, pooling a specific volume for each of the 65 *ATM* amplicons. Subsequent 454 emulsion PCR and amplicon sequencing were performed with the Genome Sequencer FLX System instrument (Roche Applied Science), following the manufacturer’s recommendations.^3,4^

Libraries of *TP53*, *NOTCH1*, *SF3B1*, *MYD88, FBXW7* and *XPO1* genes were separately generated, pooling 31 amplicons for 3 patients. Furthermore, libraries of *BIRC3* gene were generated pooling 11 amplicons for 8 patients. In these cases, subsequent Agencourt AMPure XP bead purification was needed to remove short fragments. Sequencing runs of these genes were carried out on a Genome Sequencer Junior System instrument (Roche Applied Science).

All sequencing data were generated using the GS FLX and Junior Sequencer Instrument software version 2.7 (Roche Applied Science). Sequence alignment and variant detection were performed using the GS Amplicon Variant Analyzer software version 2.7 (Roche Applied Science). The results were further processed and visualized using the Sequence Pilot software version 3.5.2 (JSI Medical System, Kippenheim, Germany).^5^ To detect variants, filters were set to display sequence variants occurring in more than 2% of bidirectional reads per amplicon in at least one patient. S7 Table shows the median number of reads generated for each gene, allowing variants to be identified down to a detection limit of 2%.^4^

All variants were first compared with published single nucleotide polymorphism data (<http://www.ncbi.nlm.nih.gov/projects/SNP/>). Mutations within introns were not scored. Mutations in coding regions were compared with previously described mutations in the COSMIC database (<http://cancer.sanger.ac.uk/cancergenome/projects/cosmic/>).^6^ In addition, all missense, frameshift and nonsense mutations were resequenced on unamplified DNA to rule out artifacts arising from whole-genome amplification. For *ATM*, mutations were defined as pathogenic if they were “truncating” due to a sequence alteration that was predicted to terminate the protein prematurely, or “non-truncating” if they were missense and predicted to be damaging by the SIFT algorithm.^7^

1. Wang L, Lawrence MS, Wan Y, Stojanov P, Sougnez C, Stevenson K, et al. SF3B1 and other novel cancer genes in chronic lymphocytic leukemia. N Engl J Med. 2011;365(26):2497-506.

2. Margulies M, Egholm M, Altman WE, Attiya S, Bader JS, Bemben LA, et al. Genome sequencing in microfabricated high-density picolitre reactors. Nature. 2005;437(7057):376-80.

3. Kohlmann A, Grossmann V, Klein H-U, Schindela S, Weiss T, Kazak B, et al. Next-generation sequencing technology reveals a characteristic pattern of molecular mutations in 72.8% of chronic myelomonocytic leukemia by detecting frequent alterations in TET2, CBL, RAS, and RUNX1. J Clin Oncol. 2010;28(24):3858-65.

4. Grossmann V, Roller A, Klein H-U, Weissmann S, Kern W, Haferlach C, et al. Robustness of amplicon deep sequencing underlines its utility in clinical applications. J Mol Diagn. 2013;15(4):473-84.

5. Klein H-U, Bartenhagen C, Kohlmann A, Grossmann V, Ruckert C, Haferlach T, et al. R453Plus1Toolbox: an R/Bioconductor package for analyzing Roche 454 Sequencing data. Bioinforma Oxf Engl. 2011;27(8):1162-3.

6. Forbes SA, Bindal N, Bamford S, Cole C, Kok CY, Beare D, et al. COSMIC: mining complete cancer genomes in the Catalogue of Somatic Mutations in Cancer. Nucleic Acids Res. 2011;39(Database issue):D945-50.

7. Kumar P, Henikoff S, Ng PC. Predicting the effects of coding non-synonymous variants on protein function using the SIFT algorithm. Nat Protoc. 2009;4(7):1073-81.

**Supplementary Data**

**Table A. Characteristics of the series of 197 CLL patients with 11q deletion.**

| **Characteristic** | **Number of patients**  **(n = 197)** | **%** |
| --- | --- | --- |
| Age, years  Median  Range | 65  28-97 |  |
| Gender  Male  Female | 151  46 | 77  23 |
| Binet clinical stage  A  B  C | 121  58  18 | 61  30  9 |
| Lymphadenopathy  No  Yes* | 62  135 | 32  68 |
| Splenomegaly (n = 192)  No  Yes | 147  45 | 77  23 |
| Hepatomegaly (n = 192)  No  Yes | 174  18 | 91  9 |
| Lymphocyte count (> 20,000/µL) (n = 192)  Yes  No | 89  103 | 46  54 |
| Serum LDH (n = 187)  Normal  High | 133  54 | 71  29 |
| Serum β2 microglobulin (n = 170)  Normal  High | 116  54 | 68  32 |
| *IGHV* mutational status (n = 56)  Unmutated (<2%)  Mutated (≥2%) | 37  19 | 66  34 |
| CD38 expression (n = 131)  Negative (<30%)  Positive (≥30%) | 59  72 | 45  55 |
| ZAP-70 expression (n = 79)  Negative (<20%)  Positive (≥20%) | 35  44 | 44  56 |
| FISH alterations in addition  11q- only  11q- and other(s)  13q-  +12  17p- | 82  115  108  14  6 | 42  58  55  7  3 |
| First therapy  Yes  No | 151  46 | 77  23 |
| Median TFT** (months) | 25 |  |
| Survival  Yes  No | 131  60 | 69.5  30.5 |
| Median OS (months) | 106 |  |

* ≥2 territories (extended lymphadenopathy): 75 patients (38.1 %). **TFT: Time to first therapy. ***OS: Overall survival.

**Table B. Univariate analysis of time to first therapy in 11q- CLL patients with respect** to the number of losses detected by FISH: <40% (n = 51) or ≥40% (n = 146).

| **Variable** | **Median time to first therapy, months** | ***P* (log-rank test)** |
| --- | --- | --- |
| Sex, male/female | 27/23 | **0.270** |
| Binet A *vs* B *vs* C | 33/10/10 | **0.024** |
| Lymphadenopathy  No *vs* ≤2 node areas *vs* >2 node areas | 45/33/10 | **< 0.0001** |
| Splenomegaly, yes/no | 8/31 | **0.045** |
| Lymphocytes >20 x 10^9^/L, yes/no | 15/33 | **0.220** |
| Serum LDH high/normal | 15/30 | **0.045** |
| β_2_ microglobulin high/normal | 12/29 | **0.019** |
| CD38 expression, high/normal | 23/38 | **0.023** |
| ZAP70 expression, high/normal | 24/45 | **0.025** |
| *IGHV* mutation status  Unmutated/mutated | 10/52 | **<0.0001** |
| del(11q) as sole cytogenetic aberration, yes/no | 22/29 | **0.550** |
| del(11q) and del(13q), yes/no | 31/20 | **0.250** |
| del(11q) <40% vs ≥40% | 44/18 | **<0.0001** |

**Table C. Univariate analysis of overall survival in 11q- CLL patients** with respect to the number of losses detected by FISH: <40% (n = 51) or ≥40% (n = 146).

| **Variable** | **Median overall survival, months** | ***P* (log-rank test)** |
| --- | --- | --- |
| Sex, male/female | 108/90 | **0.680** |
| Binet A *vs* B *vs* C | 119/74/62 | **0.001** |
| B symptoms, yes/no | 77/108 | **0.034** |
| Lymphadenopathy  No *vs* ≤2 node areas *vs* >2 node areas | 132/92/85 | **0.087** |
| Splenomegaly, yes/no | 68/119 | **< 0.0001** |
| Hepatomegaly, yes/no | 68/108 | **0.025** |
| Lymphocytes >20 x 10^9^/L, yes/no | 83/119 | **0.032** |
| Serum LDH high/normal | 67/108 | **< 0.0001** |
| β_2_ microglobulin high/normal | 66/132 | **< 0.0001** |
| CD38 expression, high/normal | 90/109 | **0.155** |
| ZAP70 expression, high/normal | 106/152 | **0.133** |
| *IGHV* mutation status  Unmutated/mutated | 106/122 | **0.818** |
| Del11q+Del13q, yes/no | 85/108 | **0.045** |
| Del11q as sole cytogenetic aberration, yes/no | 88/106 | **0.425** |
| Del11q <40% *vs* ≥40% | 90/NR* | **0.006** |

*NR: not reached

**Table D. Main clinical and biological characteristics of 25 CLL patients with 11q- with respect to *ATM*** mutational status.

| **Variable** | **Category** | **Subgroup with *ATM* mutations (n=9)** | **Subgroup without *ATM* mutations n=16)** | ***P*** |
| --- | --- | --- | --- | --- |
| Gender | Male | 6/8 (75%) | 14/17 (82.4%) | **0.525** |
| Age (years), median (range) |  | 69 (48-83) | 70 (29-98) | **0.789** |
| Binet stage | A | 5/8 (62.5%) | 10/17 (58.8%) | **0.607** |
|  | B or C | 3/8 (37.5%) | 7/17 (41.2%) |  |
| White blood cells (x 10^9^/L), median (range) |  | 19945 (12200-190900) | 19600 (5960-91000) | **0.56** |
| Lymphocytes (x 10^9^/L), median (range) |  | 14520 (7200-177537) | 14280 (5010-80860) | **0.485** |
| Hemoglobin (x 10^9^/L), median (range) |  | 14.9 (10.4-16.8) | 13.7 (6.8-18.7) | **0.366** |
| Platelet (x 10^9^/L), median (range) |  | 207000 (151000-269000) | 173000 (108000-257000) | **0.062** |
| *IGHV* | unmutated | 8/8 (100%) | 11/14 (78.6%) | **0.236** |
| LDH | >UNL | 2/7 (28.6%) | 5/17 (29.4%) | **0.682** |
| β2 microglobulin | >UNL | 1/5 (20%) | 4/14 (28.6%) | **0.603** |
| LDT | <1 year | 1/6 (16.7%) | 7/14 (50%) | **0.187** |
| Bone marrow pattern | Diffuse | 2/5 (40%) | 2/9 (22.2%) | **0.455** |
| Hepatomegaly | Yes | 2/7 (28.6%) | 1/16 (6.3%) | **0.209** |
| Splenomegaly | Yes | 2/7 (28.6%) | 4/16 (25%) | **0.618** |
| B symptoms | Yes | 0/8 (0%) | 2/17 (11.8%) | **0.453** |
| Died during follow-up | Yes | 4/8 (50%) | 4/17 (23.5%) | **0.193** |
| Therapy during follow-up | Yes | 7/8 (87.5%) | 11/17 (64.7%) | **0.246** |

*IGHV* unmutated: ≥98% homology with germline; LDH: lactate dehydrogenase; UNL: upper normal level; LDT: lymphocyte doubling time

**Table E**. PCR primers used for next-generation sequencing studies.

| **Gene** | **Strand** | **Exon** | **Amplicon** | **Forward Sequence 5' -> 3'** | **Reverse Sequence 5' -> 3'** | **Length (bp)** |
| --- | --- | --- | --- | --- | --- | --- |
| *ATM* | + | 2 | 1 | TGATGTGTGTTCTGAAATTGTGA | ACACACAAAAGTAATATCACAACAGAA | 369 |
| *ATM* | + | 3 | 2 | TGCCGTCAACTAGAACATGATAG | TGCCAAATTCATATGCAAGG | 370 |
| *ATM* | + | 4 | 3 | GCTCTTTGTGATGGCATGAA | AACAAACTTATGCAACAGTTAAGTCC | 342 |
| *ATM* | + | 5 | 4 | GCCATAATTTGCCAATTTCTTC | GACAGAGTGCTTTCTTTGGTGA | 380 |
| *ATM* | + | 6 | 5 | CCTTTTTCTGTATGGGATTATGGA | TTACTGAGTCTAAAACATGGTCTTGC | 345 |
| *ATM* | + | 7 | 6 | CCCCCTGTTATACCCAGTTG | TCAACCAGAGAAATCCAGAGG | 396 |
| *ATM* | + | 8 | 7 | AGGTTGGACCAGGTGTCTTC | CAGGAAATTTCTAAATGTGACATGA | 322 |
| *ATM* | + | 9 | 8 | GTGCTGTTCCACTCCAACCT | GGTTGAGATGAAAGGATTCCAC | 352 |
| *ATM* | + | 10 | 9 | ATGGAAATGATGGTGATTCTCTAAT | TGCTCAGAACTTATACCACGAAAG | 380 |
| *ATM* | + | 10 | 10 | GGTCAAACCTAGAAAGCTCACAA | AAGTCTTCTCGGCCAAACAA | 354 |
| *ATM* | + | 11 | 11 | GCCAGGCACTGTCCTGATA | AAAGCCATCTGGCATCAAAT | 357 |
| *ATM* | + | 12 | 12 | CAATGGTTGTCCTCCTTAAATTG | TAAGATGCAGCTACTACCCAGCTA | 370 |
| *ATM* | + | 13 | 13 | AGGCAAAGCATTAGGTACTTGG | TTCTCCTTCCTAACAGTTTACCAAAG | 348 |
| *ATM* | + | 14 | 14 | CCAGGATATGCCACCTTTAACT | TTTCATTCAAATTTATCCGAAAC | 308 |
| *ATM* | + | 15 | 15 | TTGCTTATACTGTATGACTACGTGGA | ACTCCAGCCTGGGTGACA | 361 |
| *ATM* | + | 16 | 16 | TTGTTGCTTGGTTCTTTGTTTG | CGTGTTAGCCAGGATGGTCT | 355 |
| *ATM* | + | 17 | 17 | ACAGATGTGAGCCACTGTGC | GGCCTCTTATACTGCCAAATCA | 340 |
| *ATM* | + | 18 | 18 | ATATGGCTGTTGTGCCCTTC | TCTTCAAAGACACCATGTGATTC | 350 |
| *ATM* | + | 19 | 19 | CTCCCAAAGTGCTGGGATTA | CATCTTGGTCACGACGATACA | 337 |
| *ATM* | + | 20 | 20 | GCCAATGGAAGATGTTCTTGA | GCATTCGTATCCACAGATAGCA | 357 |
| *ATM* | + | 21 | 21 | TGGCAAGGTGAGTATGTTGG | TGGCCCATTTTGAATAAGGA | 370 |
| *ATM* | + | 22 | 22 | CAGGCATCTAACAAAGGAGAGG | TACTGCCATCTGCAGCATTC | 374 |
| *ATM* | + | 23 | 23 | TGCTTTGGAAAGTAGGGTTTG | TGATTTGACCCATTGTGACC | 375 |
| *ATM* | + | 24 | 24 | TCTGGAGTTCAGTTGGGATTTTA | AAGTGCCACTCAGAAAATCTAGC | 346 |
| *ATM* | + | 25 | 25 | AGTTGAATGAATGTTGTTTCTAGGTC | TGGTATGTGTGTTGCTGGTG | 369 |
| *ATM* | + | 26 | 26 | TTATGGTGGTGGTATGTTCTAAGC | GAAGACTTTTCCAGTCCTCTTGA | 320 |
| *ATM* | + | 26 | 27 | GATGAGGTGAAGTCCATTGCT | AATTCATGGGTTGGCTATGC | 348 |
| *ATM* | + | 27 | 28 | TGCCTTTTGAGCTGTCTTGA | CCTCAATTCAAAGGTGGCTATT | 335 |
| *ATM* | + | 28 | 29 | GAAGTTCACTGGTCTATGAACAAAAC | CAATTAATGCTGACAAGTAAAATACCA | 368 |
| *ATM* | + | 29 | 30 | TTGTAGCCGAGTATCTAATTAAACAAG | GCGGACAGAGTGAGTCTTTG | 360 |
| *ATM* | + | 30 | 31 | GAGATGCTGAACAAAAGGACTTC | CCAAATAAACATAAAACACTCAAATCC | 354 |
| *ATM* | + | 31 | 32 | TGGCTTACTTTAAAATTATTTCTCTCC | CCAATGTGCCTGGCCTAC | 364 |
| *ATM* | + | 32 | 33 | TCACAGGCTTAACCAATACGTG | CAGGAAGAGTTCAGGGTGAGA | 370 |
| *ATM* | + | 33 | 34 | TTTCTCTGTTGTCACATATTGCTAATC | ACGCAGCGCATGACTGTA | 340 |
| *ATM* | + | 34 | 35 | TCGGCCTTAAGGTTAATTCTTG | TGTGTGAAGTATCATTCTCCATGA | 362 |
| *ATM* | + | 35 | 36 | TGGTGTACTTGATAGGCATTTGA | TCTGAGCTTTTCCACACTGC | 377 |
| *ATM* | + | 36 | 37 | AGGAAAGGTACAATGATTTCCACT | TGAAGATGATGTGCAGTATCACAG | 321 |
| *ATM* | + | 37 | 38 | ATATGTCAACGGGGCATGA | TGGGATTCCATCTTAAATCCAT | 345 |
| *ATM* | + | 38 | 39 | GGTACTGCCCACCAGAACCT | CCTGAATATGGATTACTGCAAGG | 360 |
| *ATM* | + | 39 | 40 | TTCTGTTAAGCAGTCACTACCATTG | GGGAACAGGAGGCAAAATAA | 351 |
| *ATM* | + | 40 | 41 | TTGTTTGCCACCTTCATTAGTTT | GATGCTTAGTCCAGTAAGTAAATTCAG | 319 |
| *ATM* | + | 41 | 42 | GAGTTGGGAGTTACATATTGGTAATG | CCACATTGCTTCGTGTTCAT | 321 |
| *ATM* | + | 42 | 43 | CTGTGGTGGAGGGAAGATGT | TGGCTGTGTAAATATCCACCAA | 323 |
| *ATM* | + | 43 | 44 | CACCACACCCAGCTGATATTT | TGTTGTTTAGAATGAGGAGAGAGG | 353 |
| *ATM* | + | 44 | 45 | TTTGTCCTTTGGTGAAGCTATTT | ACATCTGATCACAGCCACCA | 324 |
| *ATM* | + | 45 | 46 | AGTAGCAAAGCCTATGATGAGAAC | GCTAGGATTACAGGCATGAGC | 340 |
| *ATM* | + | 46 | 47 | TCATTTCTCTTGCTTACATGAACTC | AAAGGAAAGTCAAGAGGTAAGATGAC | 324 |
| *ATM* | + | 47 | 48 | GTGGGGAGATGTCATGCAG | CAGTAAAACACTAATCCAGCCAAT | 364 |
| *ATM* | + | 48 | 49 | GGGCAGTTGGGTACAGTCAT | TTTCAAAATTGGCTGCTTTG | 368 |
| *ATM* | + | 49 | 50 | ATTTAAATTGGTTGTGTTTTCTTGA | ATATATAGTTAAGCCGACCTTTAGAGC | 376 |
| *ATM* | + | 50 | 51 | CCCTTTATAATCCTTAGAAGTTTGC | GGGTAGAATATTGGGCTGAGTAAC | 380 |
| *ATM* | + | 51 | 52 | TGAATCCAGTTTAATTTAGGACCAA | GAAATCCTAGGCCTCCCATC | 333 |
| *ATM* | + | 51 | 53 | GAATGGGGACCAAGATGATG | TGCAGTGGGTAGAGCGTGTA | 273 |
| *ATM* | + | 52 | 54 | TGTTAAGCAAAATGAAAAATATGGA | CCAGCCTTGAACCGATTTTA | 348 |
| *ATM* | + | 53 | 55 | GAGAAGTTTAAATGTTGGGTAGTTCC | AAAGTAACCAGGGAATGCTGAA | 355 |
| *ATM* | + | 54 | 56 | CAATCAGAGCCTGAACCACA | GGCTTGGGCAAAGGAAATA | 370 |
| *ATM* | + | 55 | 57 | TCACATCGTCATTTGTTTCTCTG | AAGACAAAATCCCAAATAAAGCAG | 350 |
| *ATM* | + | 56 | 58 | ATTGGTTTGAGTGCCCTTTG | CTGAGGCTGGTCTCGAACTC | 358 |
| *ATM* | + | 57 | 59 | GCTGAATGATCATCAAATGCTCT | TTTGACAATTACCTGATGAAATTAAAG | 336 |
| *ATM* | + | 58 | 60 | TGCTTCCCTGTCCAGACTGT | TGCCAAACAACAAAGTGCTC | 366 |
| *ATM* | + | 59 | 61 | AAACTCAACATGGCCGGTTA | TTGGTAGGCAAACAACATTCC | 320 |
| *ATM* | + | 60 | 62 | CATGTGGTTTCTTGCCTTTG | ACTCAAACTCCTGGGCTCAA | 348 |
| *ATM* | + | 61 | 63 | AACGTCTAATGAAAGCCCACTC | TGAAGCAGTGCTCTTCACATC | 326 |
| *ATM* | + | 62 | 64 | GGAAACATGAAGTGTGCATGA | CACAAAGGTTTCAGTGAGGTGA | 377 |
| *ATM* | + | 63 | 65 | GCAGATGACCAAGAATGCAA | TCTAAAGGCTGAATGAAAGGGTA | 361 |
| *NOTCH1* | - | 33 | 1 | CCGTAGATGACCTGGGTGAG | CGAGACCCTGTGGGTCAG | 338 |
| *NOTCH1* | - | 34 | 2 | ACTGCTGCTTCCTCTGGTGA | CTTGAGGCTGCCCAGGTA | 358 |
| *NOTCH1* | - | 34 | 3 | AGGCTGCTGGACGAGTACAA | CACGGACGGAGACTGCTG | 357 |
| *NOTCH1* | - | 34 | 4 | CCATGGCTACCTGTCAGACG | TATTGGTTCGGCACCATGC | 351 |
| *NOTCH1* | - | 34 | 5 | GAATGGTCAATGCGAGTGG | CTGAGCTCACGCCAAGGT | 349 |
| *NOTCH1* | - | 34 | 6 | TGCAGCCACAAAACTTACAGA | TGTCCACAGGCGAGGAGTAG | 333 |
| *NOTCH1* | - | 34 | 7 | CTGCCATCCTCGCTGGT | ATCCACAGAGCGCACACA | 335 |
| *SF3B1* | - | 10 | 1 | GTGCAAATATTGTTCATTATGCTGT | TGTTAAGGGAAGTTGAAATGTTATGA | 345 |
| *SF3B1* | - | 11 | 2 | TCATAACATTTCAACTTCCCTTAACA | ACATGGCCAGGTGCAGAG | 331 |
| *SF3B1* | - | 12 | 3 | TGGAAAATCTTCTTTGAGTAATTTG | GTGCAAAGGAAAAGGTCTAGGA | 364 |
| *SF3B1* | - | 13 | 4 | TCTTAAACAGTTCGTCCCTTGA | GTAGCCAGACCAGCAGCCTA | 360 |
| *SF3B1* | - | 14 | 5 | CCAACTCATGACTGTCCTTTCT | CATAGTAAGACCCTGTCTCCTAAAGA | 369 |
| *SF3B1* | - | 15 | 6 | TGAGAGAATCTGGATGATATTGTGT | TTCAAGAAAGCAGCCAAACC | 336 |
| *SF3B1* | - | 16 | 7 | GCAACTCCTTATGGTATCGAATC | GAACCATGAAACATATCCAGTTTACA | 369 |
| *TP53* | - | 4 | 1 | ACCTGGTCCTCTGACTGCTC | CAGGCATTGAAGTCTCATGG | 361 |
| *TP53* | - | 5 | 2 | CACTTGTGCCCTGACTTTCA | CACTCGGATAAGATGCTGAGG | 343 |
| *TP53* | - | 6 | 3 | CAGATAGCGATGGTGAGCAG | TTGCACATCTCATGGGGTTA | 335 |
| *TP53* | - | 7 | 4 | GCACTGGCCTCATCTTGG | AAGAGGTCCCAAAGCCAGAG | 334 |
| *TP53* | - | 8 | 5 | GGACAGGTAGGACCTGATTTC | TCTCCATCCAGTGGTTTCTTC | 346 |
| *TP53* | - | 9 | 6 | AAAGGGGAGCCTCACCAC | TGTCTTTGAGGCATCACTGC | 342 |
| *TP53* | - | 10 | 7 | GCTGTATAGGTACTTGAAGTGCAG | CTGCCTTTGACCATGAAGG | 349 |
| *TP53* | - | 11 | 8 | AGGGAAAAGGGGCACAG | CCCCACAACAAAACACCAGT | 339 |
| *FBXW7* | - | 8 | 1 | TTACTGTTCCTGTTTATGCCTTCA | TGAGCTCTGATATTTGCTATCCA | 341 |
| *FBXW7* | - | 9 | 2 | TCTGAAGAGCCAAACAAATTAAATAAG | GGCCCAAATTCACCAATAATAG | 360 |
| *FBXW7* | - | 10 | 3 | TTGACTAAATCTACCATGTTTTCTCA | TTCATCAGGAGAGCATTTAAGG | 359 |
| *FBXW7* | - | 11 | 4 | GGTCAGTAATTGATAGGAAGAGTATCC | TGATGCTAAGGCTCCATATTTC | 349 |
| *FBXW7* | - | 12 | 5 | AACTCATAGCCATTATTTCTAACCAG | GGAGTATATCGTCTACACAATTGGA | 369 |
| *MYD88* | + | 4 | 1 | GCCCTTCCTGAAGCTATTCC | CCTGGCACACATTCACTCAC | 347 |
| *MYD88* | + | 5 | 2 | GTTGAAGACTGGGCTTGTCC | GAAGTTGGCATCTCCAGGAA | 354 |
| *XPO1* | - | 14 | 1 | GCTCATTATTTGCATTTGAAACC | CTTTGCCTCTTTTCTGTTCACAT | 343 |
| *XPO1* | - | 15 | 2 | TGTGAACAGAAAAGAGGCAAAG | CCTGAACCTGAACGAAATGC | 355 |
| *BIRC3* | + | 2 | 1 | AAGCAAAGCCATGCACAAA | CGAATCTGCAGCTAGGATACAA | 356 |
| *BIRC3* | + | 2 | 2 | GTGGCCTGATGCTGGATAAC | GTCAATGGCCATGTCTGAAA | 340 |
| *BIRC3* | + | 2 | 3 | CCACTGTGCAATGAATAACGA | CTTGCAAGCTGCTCAGGATT | 348 |
| *BIRC3* | + | 3 | 4 | AGCATGCAGACACATGCAG | CAAGAATTTGCAAAGAGAATTTCA | 340 |
| *BIRC3* | + | 4 | 5 | TGATTTGAGGCAAACAACCA | CTTTCTAAAATCATGTCAGGTTTGAT | 351 |
| *BIRC3* | + | 5 | 6 | CCTTAATGAAAATGGGTTTTGTT | TTCAATATTAACAGTACCAAATGACTC | 360 |
| *BIRC3* | + | 6 | 7 | CCAAGTTGTTATTTAATGACTGAACG | TCTATAATTCTCTCCAGTTGCTAGGAT | 349 |
| *BIRC3* | + | 6 | 8 | GCAATCATGATGAATACTCCTGT | TTGAATTCAGAATACTAAACATGAAAA | 346 |
| *BIRC3* | + | 7 | 9 | GGAAGTTTGTGAGCAGAGTTTG | AAGAGCGTATTTTCAATTGACTTAGA | 367 |
| *BIRC3* | + | 8 | 10 | CCTTGAAATGAGTATTTGGCTA | CCACAAGGAATAAACACTATGGA | 358 |
| *BIRC3* | + | 9 | 11 | GAAGAAAGAACATGTAAAGTGTGTATG | CAAAAGCCTATCATTCTCTTAGTTTC | 359 |

**Table F. A: PCR amplification protocol for *ATM***.

| **Cycles** | **Time (min)** | **Temperature (°C)** |
| --- | --- | --- |
| 1 | 05:00 | 95.0 |
| 10 | 00:30 | 95.0 |
|  | 00:30 | 63.0 → 58.0 |
|  | 00:30 | 72.0 |
| 28 | 00:30 | 95.0 |
|  | 00:30 | 58.0 |
|  | 00:30 | 72.0 |
| 1 | 07:00 | 72.0 |

B: PCR amplification protocol for the remaining genes.

| **Cycles** | **Time (min)** | **Temperature (°C)** |
| --- | --- | --- |
| 1 | 10:00 | 95.0 |
| 10 | 00:30 | 95.0 |
|  | 00:30 | 63.0 → 58.0 |
|  | 00:30 | 72.0 |
| 25 | 00:30 | 95.0 |
|  | 00:30 | 58.0 |
|  | 00:30 | 72.0 |
| 1 | 07:00 | 72.0 |

**Table G**. Median frequency of reads generated by next-generation sequencing (NGS).

| **Gene** | **Median of reads generated by NGS** |
| --- | --- |
| *ATM* | 890 (151-6608) |
| *TP53* | 1037(263-3743) |
| *NOTCH1* | 635 (90-2314) |
| *SF3B1* | 951 (224-3565) |
| *MYD88* | 962.5 (297-3008) |
| *FBXW7* | 1050 (233-3777) |
| *XPO1* | 945.5 (163-2635) |
| *BIRC3* | 1116 (84-4130) |

**Figure A. Time to first therapy (TFT) and B. Overall survival (OS) of the global series of 197 CLL patients with 11q deletion.**

A.


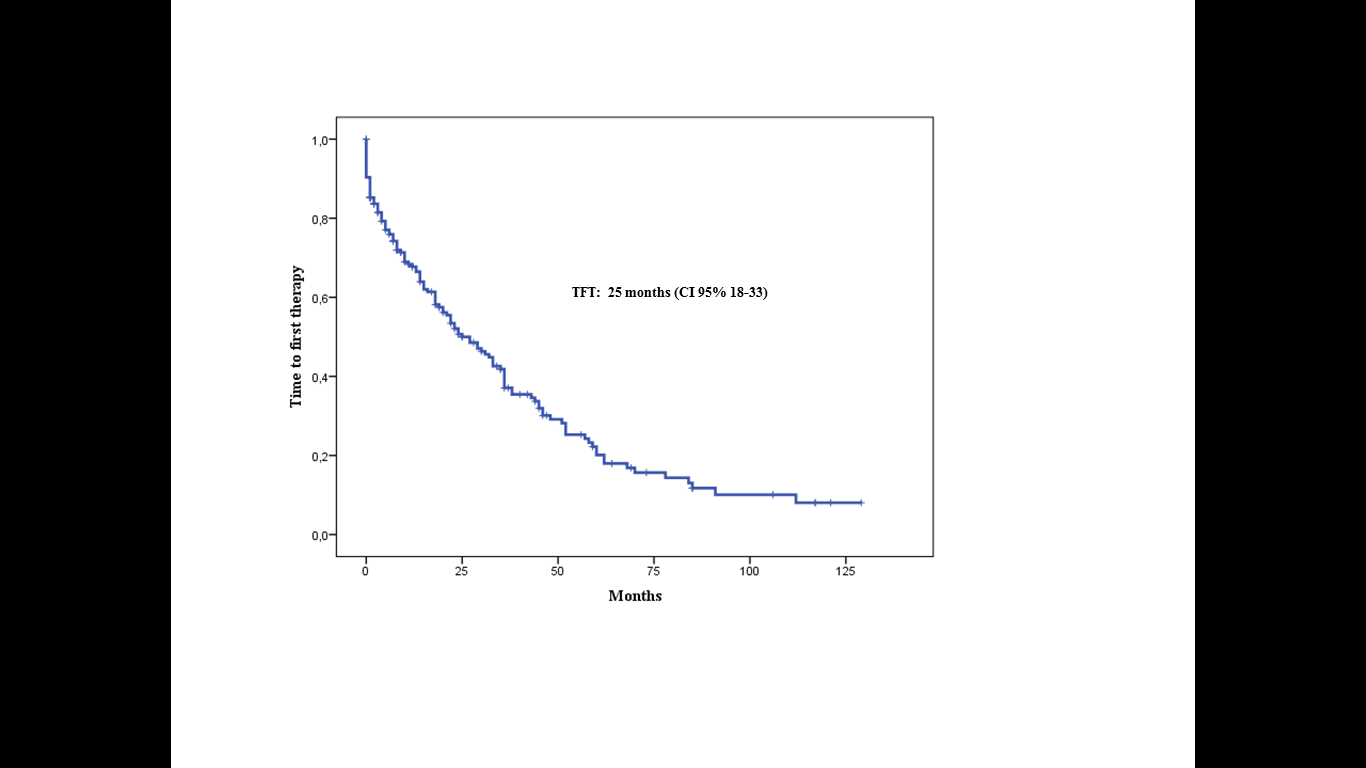


B.


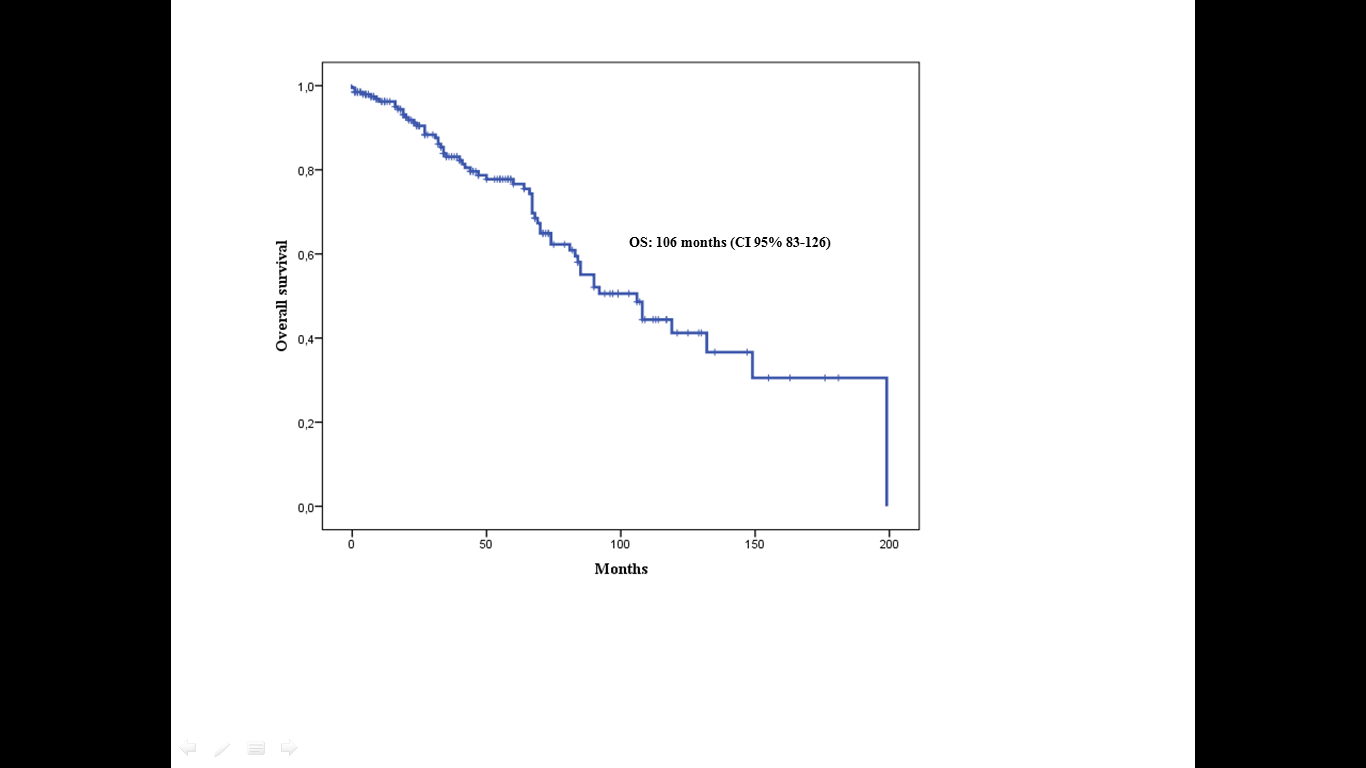


**Figure B. A. Time to first therapy (TFT) and B. Overall survival (OS) of patients with CLL and 11q deletion and a percentage of FISH losses <40%, 41-59% or ≥60%.**

A.


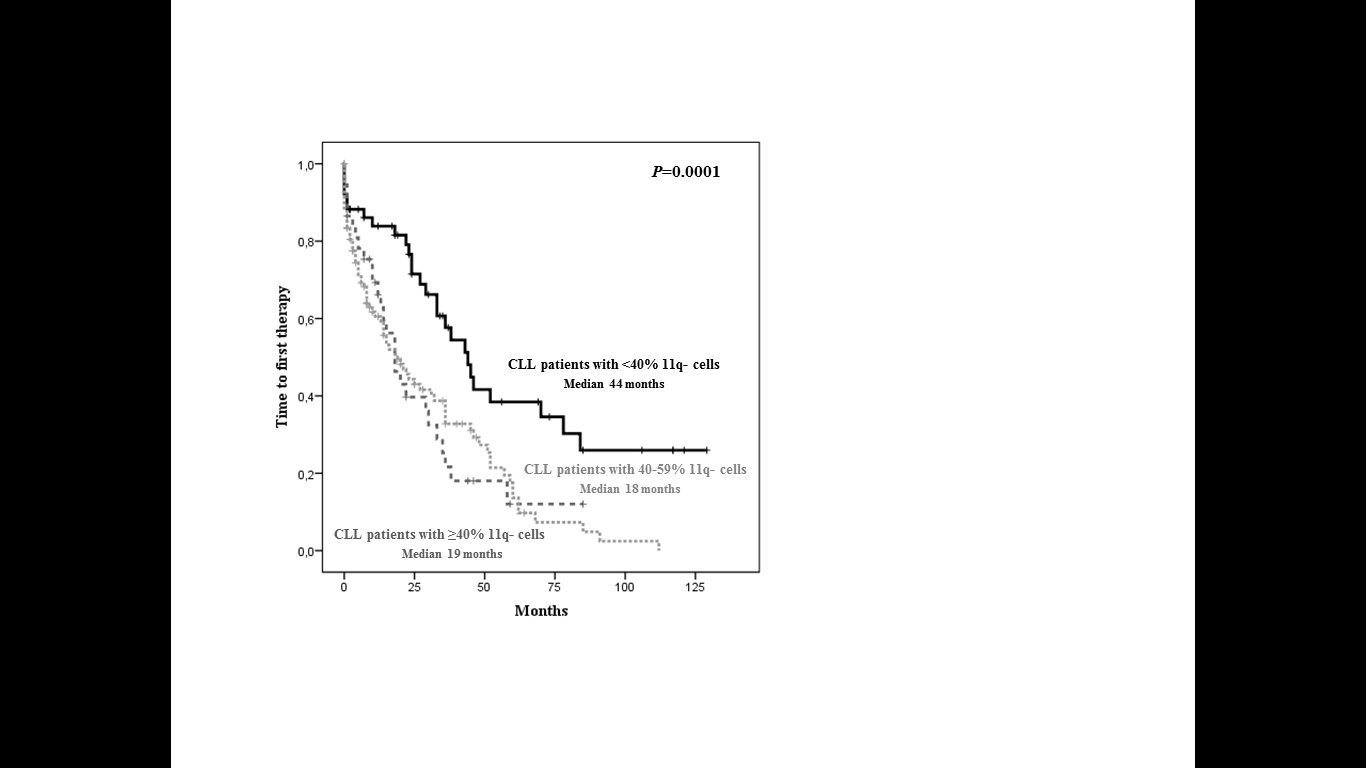


B.


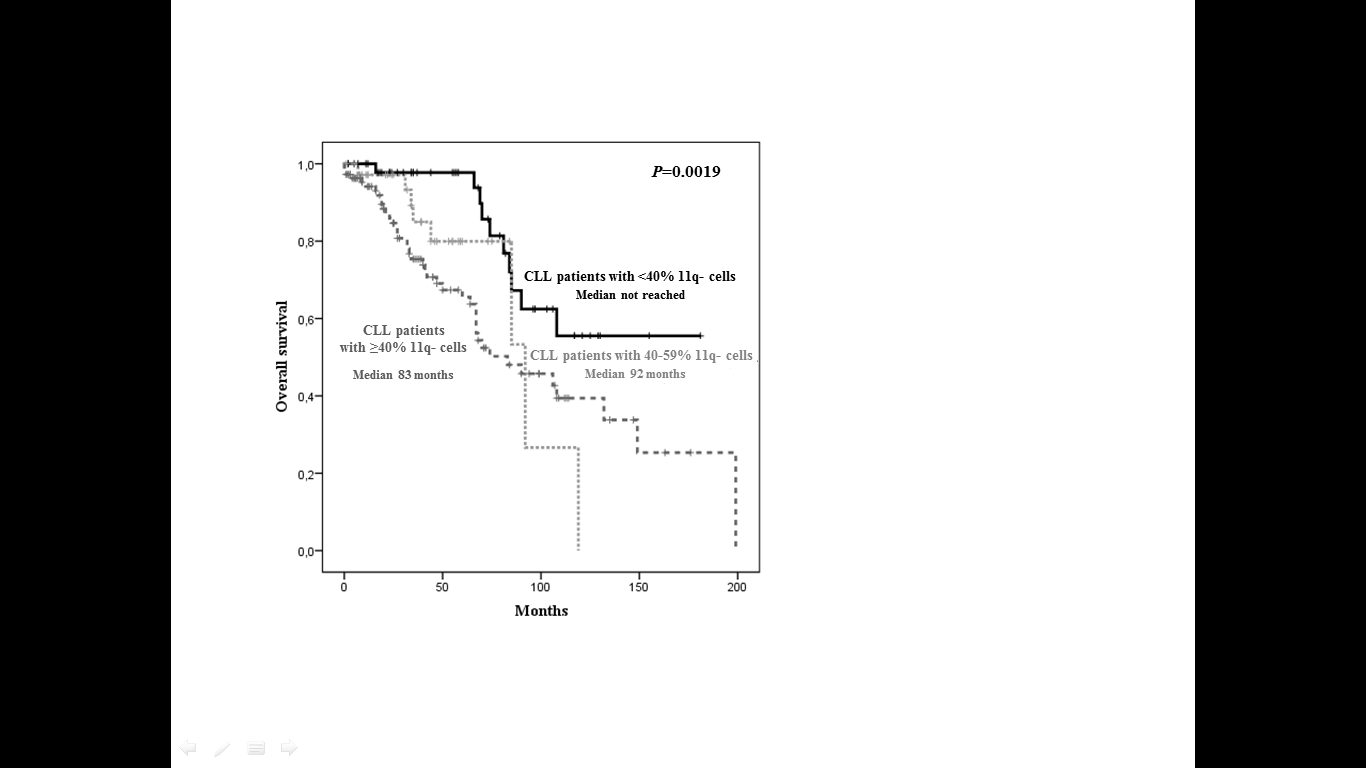


**Figure C. Kaplan-Meier plots of time to first therapy (TFT) (A) and overall survival (OS) (B) from diagnosis for 11q- CLL patients sequenced for *NOTCH1* and *TP53*, respectively.**

A.


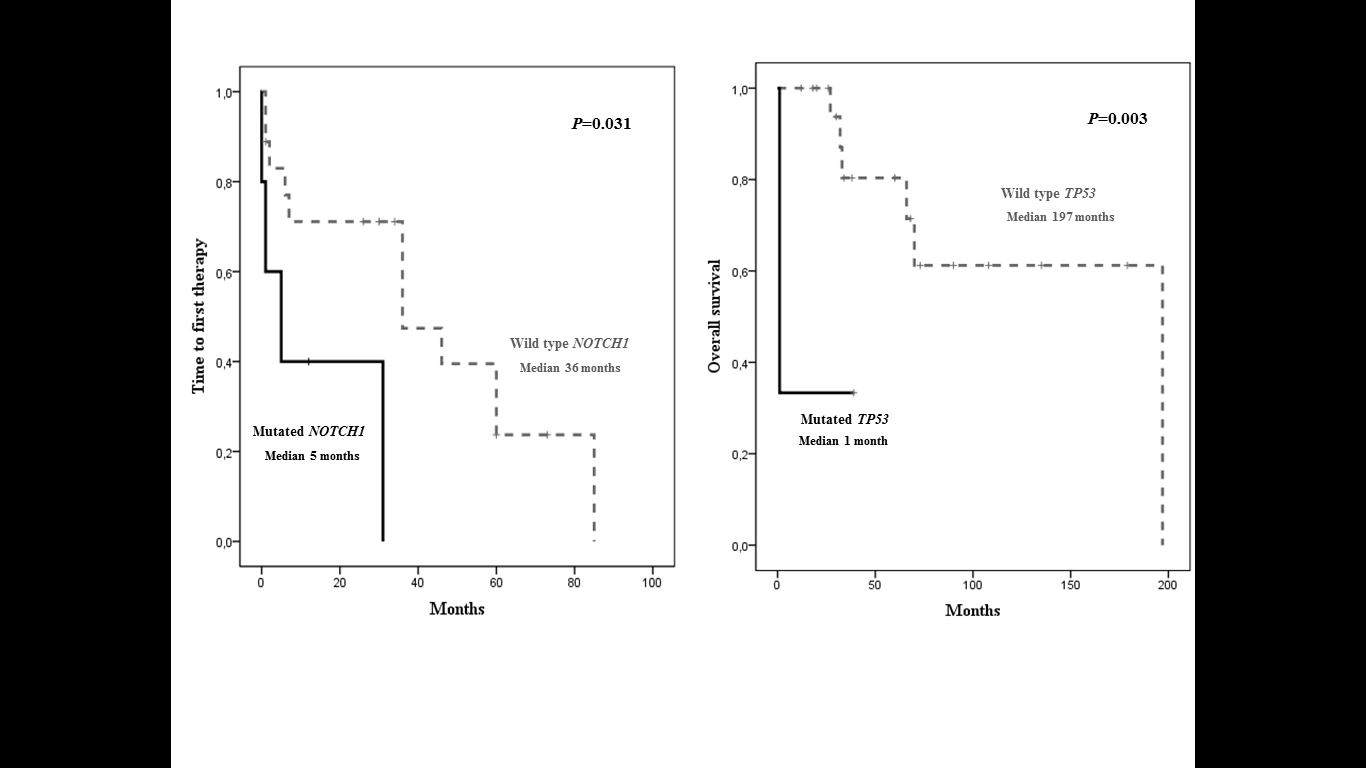


B.


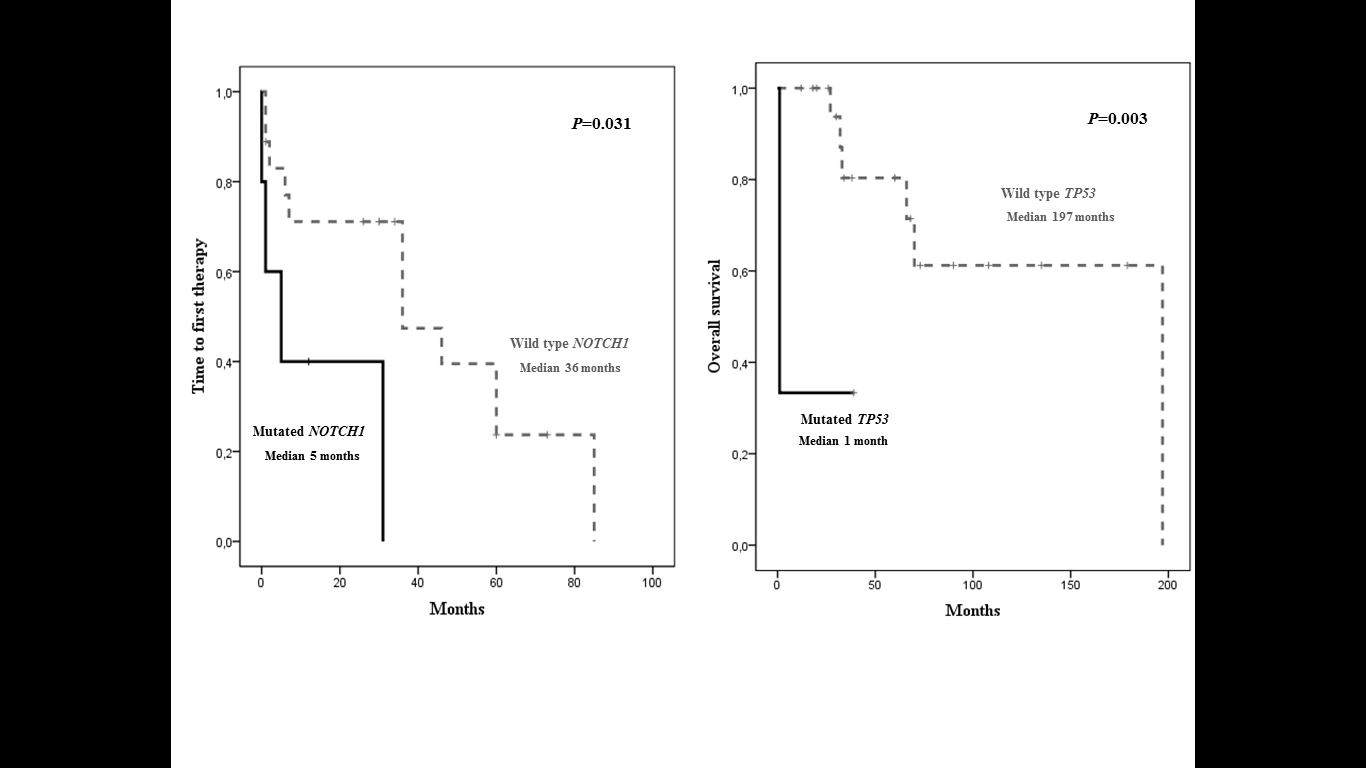

Supplement: S1 File — Characteristics of the series of 197 CLL patients with 11q deletion (Table A). Univariate analysis of time to first therapy in 11q- CLL patients with respect to the number of losses detected by FISH: <40% (n = 51) or ≥40% (n = 146) (Table B). Univariate analysis of overall survival in 11q- CLL patients with respect to the number of losses detected by FISH: <40% (n = 51) or ≥40% (n = 146) (Table C). Main clinical and biological characteristics of 25 CLL patients with 11q- with respect to ATM mutational status (Table D). PCR primers used for next-generation sequencing studies (Table E). A: PCR amplification protocol for ATM. B. B: PCR amplification protocol for the remaining genes (Table F). Median frequency of reads generated by next-generation sequencing (NGS) (Table G). A. Time to first therapy (TFT) and B. Overall survival (OS) of the global series of 197 CLL patients with 11q deletion (Fig A), A. Time to first therapy (TFT) and B. Overall survival (OS) of patients with CLL and 11q deletion and a percentage of FISH losses <40%, 41–59% or ≥60% (Fig B). Kaplan-Meier plots of time to first therapy (TFT) (A) and overall survival (OS) (B) from diagnosis for 11q- CLL patients sequenced for NOTCH1 and TP53, respectively (Fig C) (DOC) [file pone.0143073.s001.doc]
